# Impact of small farmers' access to improved seeds and deforestation in DR Congo

**DOI:** 10.1038/s41467-023-37278-2

**Published:** 2023-03-23

**Authors:** Tanguy Bernard, Sylvie Lambert, Karen Macours, Margaux Vinez

**Affiliations:** 1 Bordeaux School of Economics, Univ. Bordeaux, Pessac, 33600 France; 2 Paris School of Economics, INRAE, Paris, 75014 France; 3 World Bank, Washington, DC 20433 USA

**Keywords:** Agriculture, Environmental impact, Agroecology, Environmental economics

## Abstract

Since the 1960s, the increased availability of modern seed varieties in developing countries has had large positive effects on households’ well-being. However, the effect of related land use changes on deforestation and biodiversity is ambiguous. This study examines this question through a randomized control trial in a remote area in the Congo Basin rainforest with weak input and output markets. Using plot-level data on land conversion combined with remote sensing data, we find that promotion of modern seed varieties did not lead to an increase in overall deforestation by small farmers. However, farmers cleared more primary forest and less secondary forest. We attribute this to the increased demand for nitrogen required by the use of some modern seed varieties, and to the lack of alternative sources of soil nutrients, which induced farmers to shift towards cultivation of land cleared in primary forest. Unless combined with interventions to maintain soil fertility, policies to promote modern seed varieties may come at the cost of important losses in biodiversity.

## Abstract (web summary)

Availability of modern seed varieties in developing countries has had positive effects on households’ well-being. Here, the authors show that without support to maintain soil fertility, access to modern seed varieties increases primary forest clearance in DR Congo.

## Introduction

Approximately 75% of the population in sub-Saharan Africa remains dependent on agriculture for its livelihood. Increasing productivity and income for smallholder farmers is therefore often seen as a central part of the fight against poverty and hunger^1^. To this end, substantial investments have been made in international agricultural research and, in particular, the development, adaptation, and promotion of modern seed varieties^2^, to which large positive outcomes are attributed, albeit with significant regional differences. The Green Revolution in Asia and Latin America entailed expansion in the use of modern seed varieties in combination with chemical fertilizers and resulted in substantial increases in yields and overall production between 1960 and 2000^2^. In 1970, the agronomist Norman Borlaug was awarded the Nobel Peace Prize for his contributions to the Green Revolution^3^. In Sub-Saharan Africa, however, the diffusion of modern seed varieties remains limited to date, and agricultural yields lag far behind those in other regions^4–6^, despite substantial government investments in fertilizer subsidies in some countries^7,8^. In fact, specifics of African crops and agroecological diversity have translated into lengthier research needs and delayed the release of usable modern seed varieties in the region until the 1980s and 1990s.

Recent studies highlight the large positive effect of the diffusion of modern seed varieties on economic well-being. A 10 percentage points increase in the adoption rate of modern seed varieties is associated with a 15% increase in GDP per capita across a set of 84 low and middle-income countries^3^. Moreover, the diffusion of modern seed varieties avoided 3–5 million infant deaths by the year 2000 across 36 poor countries^9^. Country-specific quasi-experimental evidence also shows clear positive impacts on child health and nutrition outcomes^10^.

Diffusion of modern seed varieties is often also argued to contribute to forest conservation^11^. This issue is of high policy relevance as land use change is the second most important human-induced source of greenhouse gas emissions globally^12^, tropical forests support at least two-thirds of the world’s biodiversity^13^, and agricultural expansion is the most important cause of tropical deforestation and loss of biodiversity^14^. According to the “Borlaug hypothesis”, improvements in agricultural technologies can reduce the demand for new farmland—and hence deforestation—by intensifying production on the best current farmland. Borlaug^11^ estimates that the world would have needed 1.2 billion additional hectares of land to achieve the global harvest of 2000 if the cereal yields of 1950 had prevailed. While estimates on the extent of land savings differ, the adoption of modern seed varieties and broader intensification of cereal production has prevented substantial areas of land from being converted to agriculture globally^4,15,16^.

The impact of introducing yield-enhancing technologies on deforestation in a given area is, however, theoretically ambiguous. The Borlaug hypothesis builds on the assumption that output market prices decline in reaction to supply increases. But when local prices are not highly responsive to local supply conditions (for example, because demand is elastic or exogenous determinants predominate), the impact of yield-enhancing technologies may locally lead to increases in land pressure by increasing the profitability of agriculture relative to other land uses^17–20^, along the lines of the Jevons’ paradox in economics. Moreover, when elastic product demand is combined with an elastic supply of labor, even more deforestation will result^21^. The Borlaug hypothesis, moreover, assumes well-functioning markets for complementary inputs, allowing farmers to maximize returns on existing land. Where access to fertilizers is constrained, and farmers lack knowledge or resources for alternative soil fertility management practices, gains from modern varieties may be maximized by cultivating them on nitrogen-rich soils, possibly implying new land conversion and deforestation.

How these different factors interact in places of particular environmental value is an important empirical question. The question, so far, has been tackled using models and simulations^16^ or with observational data analyzing country-level trends^17,22^, within-country variation^23^, or household-level differences^24,25^. A review of this literature^26^ concludes that “the empirical evidence on a positive link between regional technological progress and deforestation is much weaker than what seems generally accepted”, though Byerlee et al.^27^ suggest that technology-driven intensification does generally correlate with land-sparing. The existing evidence is arguably not sufficient to conclude that agricultural intensification, and in particular the use of modern seed varieties, affects deforestation. Indeed, farmers who decided to clear a forested plot and use it for modern seed varieties are likely to differ from other farmers in many characteristics other than their use of modern seed varieties. Hence the impact of agriculture on deforestation may be confounded by the effect of these other—unaccounted—differences. A recent review of the literature on agriculture and deforestation in the Congo Basin forest finds no study with credible causal estimates^28^.

This paper uses a randomized controlled trial (RCT) to study the causal impact of modern seed variety diffusion on deforestation of the Democratic Republic of Congo (DRC), home to the largest share of the Congo River Basin forest, the second largest tropical forest in the world. The study setting is the former Equateur province (as defined before the 2015 administrative reform, and which now comprises the smaller Équateur province, as well as the Tshuapa, Mongala, Nord-Ubangi, and Sud-Ubangi provinces), host to a substantial proportion of the country’s forest cover. We study a large government program that provided subsidies for improved seed varieties of rice (short cycle), maize (high yielding), groundnuts (high yielding), and cassava (disease resistant). These varieties result from conventional breeding based on selection and crossbreeding by the National Agricultural Study and Research Institute in DRC, in partnership with CGIAR centers, and were selected for incorporation in the government program based on their suitability for the agro-ecological conditions of Equateur. None of the varieties is based on genetic modification. Out of the 92 villages covered by our study, 60 were randomly selected for the subsidy intervention. The remaining 32 villages served as control. In the treatment villages, households were randomly chosen ahead of the agricultural season to receive vouchers for the purchase of modern seed varieties at a subsidized price. The vouchers could be used for any of five of the main local staple crops and were redeemable (within 3 months) at the stores of local seed multipliers. As these stores tend to be located in distant urban areas, 35 of the treatment villages were selected for an additional intervention: a truck offered the seeds for sale directly in the center of the village a few days after the voucher distribution. This provides the exogenous variation needed to analyze the impact of the promotion of modern seed varieties on land use decisions.

Our first contribution is to provide unbiased evidence of the impact of modern seed variety use on deforestation based on a randomized control trial. We define deforestation as the partial or complete removal of woody vegetation in a forested area, and we combine remote-sensing-based data at the village level with household and plot-level survey data to measure deforestation. We show evidence of changes in land conversion decisions drawing on exogenous variation in both village and household access to modern seed varieties. We also test for labor reallocation, given the potentially important role of labor supply elasticity. To our knowledge, this is the first rigorous causal (experimental) evidence of the relationship between the promotion of modern seed varieties and deforestation in the Congo Basin forest.

Our second contribution is to show the importance of distinguishing between the deforestation of primary and secondary forest. Following international definitions^29,30^, we define a primary forest as a forest with no observable traces of cultivation and a secondary forest as a forest that is a more recent regrowth on land that has been previously cultivated. Because the household survey data is very granular and provides information on all plots under cultivation for a large sample of farmers, we can use it to relate the differences in subsidy levels introduced at the household level directly with their land-use decisions. We cross-validate the results by examining farmers’ reported labor allocation to land preparation on the primary and secondary forest and compare them with remote sensing-based results.

The distinction between primary and secondary forest is relevant as deforestation of primary forest has substantial implications for carbon sequestration and biodiversity^31,32^. The distinction is also immediately relevant to farmers’ decision-making, as farmers using modern seed varieties know that they have higher soil nutrient requirements than traditional varieties. Theoretically, the additional nutrients could come from mineral fertilizer, soil conservation practices, or from organic fertilizer. But when there are constraints on such soil fertility management practices (due to labor or fertilizer market imperfections and/or asymmetric information), farmers may decide their best option is to cultivate the most fertile soils and therefore shift toward the cultivation of land cleared from the primary forest (where soils are richer in nitrogen). In line with this reasoning, the risk of deforestation of primary forest is likely to be sensitive to the type of seeds provided unless seed provision is accompanied by measures to increase soil fertility.

In this work, we find that modern seed variety subsidy led to an increase in the deforestation of primary forest by beneficiary households. The increase in primary forest conversion is partly offset by a decrease in secondary forest conversion. The overall impact on deforestation (when aggregating primary and secondary forest) is not statistically significant, while the promotion of modern seed varieties of legumes is associated with less deforestation than the promotion of modern cereal seed varieties. By providing separate causal estimates for primary and secondary forest, this study provides micro-empirical evidence that can help motivate and extend the literature quantifying the implications of yield improvements and agricultural land sparing on greenhouse gas emissions, carbon sequestration, and biodiversity^33–35^.

## Results

We first document land conversion decisions in the absence of subsidies for modern varieties. Only half of the area under cultivation in the control villages for the 2014 main agricultural season was already under cultivation the year before (see control village means in Table A.6 in Supplementary Information). Of the newly cultivated area constituting the other half, 42% of the plots were in land that was previously fallow, and 58% were land that had not been recently used for smallholder production. These land conversions came from the primary forest (12%), secondary forest (72%), or from savanna and abandoned plantations (16%).

**Fig. 1:**
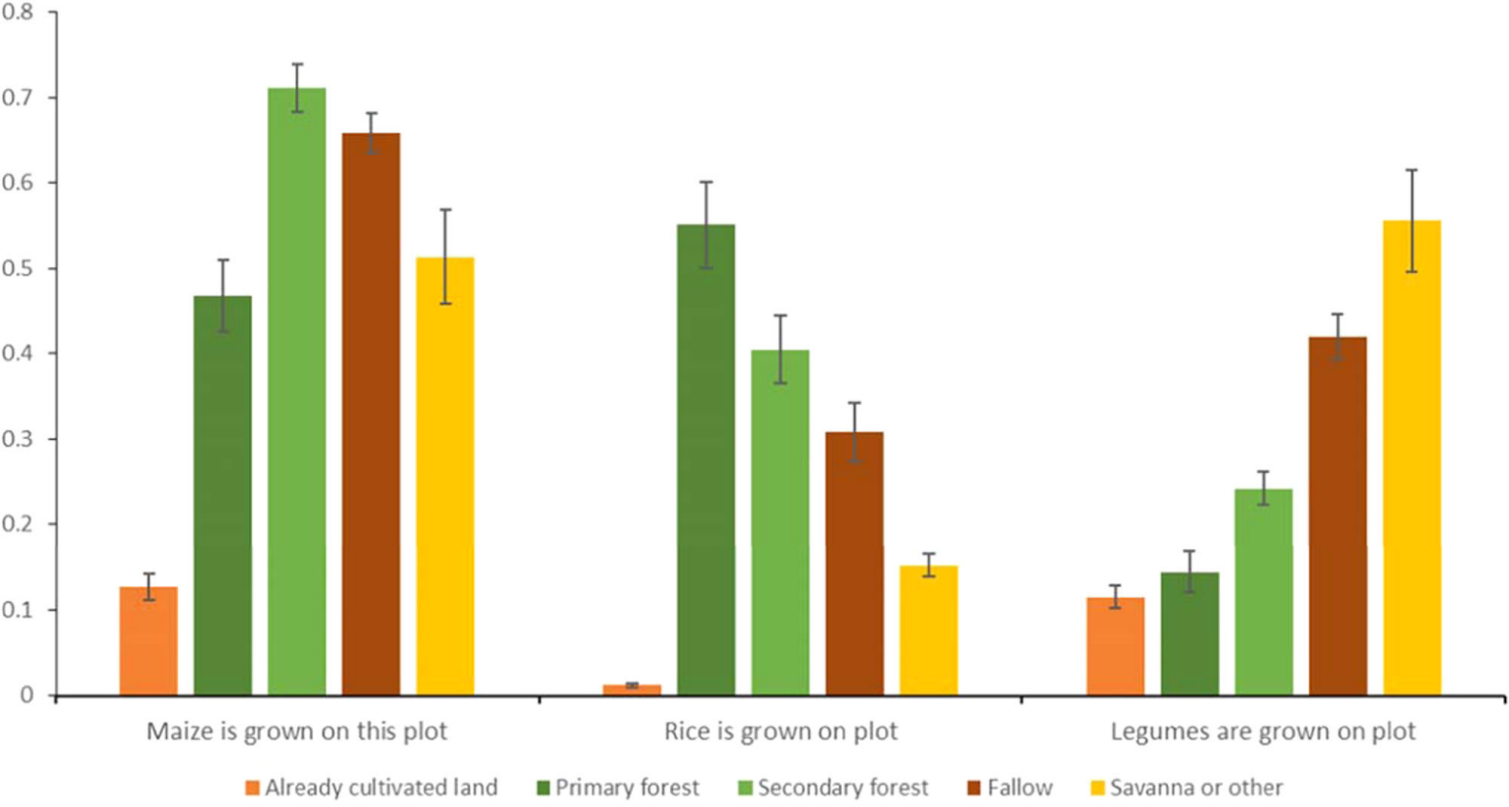
Probability of crops being grown on each type of plot. Note: Results from Ordinary Least Square regressions on a sample of *n* = 6451 independent plots (several crops can be planted on the same plot in a given year, such that columns do not add up to unity). The dependent variable measures whether the crop was grown on the plot; independent variables indicate the type of plot. Each bar represents the ordinary least square (OLS) point estimates, and vertical lines show a 95% confidence interval, with standard errors clustered at the village level. Source data are provided as a Source Data file (sourcedata.xlsx). The raw data and code used to obtain the estimates are available at https://www.openicpsr.org/openicpsr/project/177141.

To motivate the subsequent causal analysis, Fig. 1 illustrates how this pattern varies with the choice of crop cultivated. We use plot-level data obtained as part of the household survey to illustrate the probability that a given crop is grown on a particular type of plot (farmers were asked about land use in the previous season for all plots they cultivate.). Cereals are far more likely to be grown on more fertile soils, such as land freshly converted from primary or secondary forest. In contrast, legumes (groundnuts in particular) are more likely to be grown on savanna soils or plots that were previously cultivated and left to fallow.

**Fig. 2:**
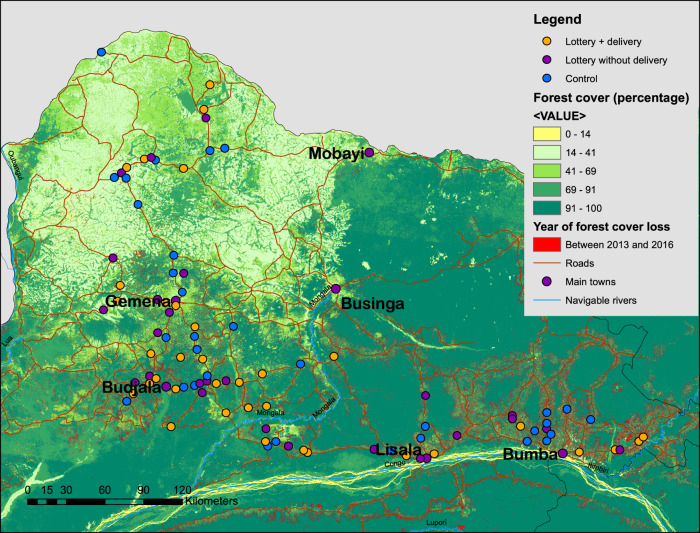
Localization of villages, forest cover, and forest cover loss in the PARRSA project area. Note: Map of PARRSA project area. Hansen forest cover is the percentage of the grid cell covered with forest according to the Hansen dataset. Localization of *n* = 92 independent villages shown on the map.

The identification of the causal effects of modern seed varieties on deforestation builds on experimental treatment variations introduced for the purpose of the study (see Methods section). The random allocation of seed subsidies created exogenous variation in access to improved varieties across 92 villages, as evidenced by Fig. 2, which shows the geographic localization of treated and control villages.

The treatment variations generated significant differences in the take-up of modern seed varieties, which are summarized in Table 1 and described in more detail in the Supplementary Methods and in Bernard et al.^36^. Compared to households receiving subsidies only, households from villages randomly assigned to receive delivery of seeds by truck were more likely to purchase seeds, but when they did, they bought smaller quantities (likely because they had only a few days to gather the necessary cash). Importantly, the truck delivery treatment is further associated with more purchase of groundnuts, while in villages without truck delivery, households were more likely to obtain cereals (rice and maize). This is consistent with the greater supply of groundnut from trucks than in the seed multipliers offices. In addition to groundnuts, households in truck delivery villages typically also bought some cereals (rice and maize). The experimental variations led to important differences in the adoption of modern seed varieties and crop choices not only in the year farmers received seed subsidies but also in the subsequent year when farmers were able to plant larger quantities from seeds recycled from the first harvest^36^.

**Table 1:** Changes in the uptake of modern seed varieties induced by experimental variations

Access	No truck delivery (30 random villages)		Truck delivery (30 random villages)	
Price subsidy	Low	High	Low	High
**Impact of intervention on:**				
Probability of buying MV^a^	Moderate	High	High	Very High
Quantity of seeds bought^b^	10 kg	10 kg	5 kg	10 kg
Most common MV seeds bought	Rice	Rice	Groundnuts	Groundnuts

^a^As compared to households in 32 randomly selected control villages.

^b^Conditional on buying modern seed variety.

### Impact of access to modern seed varieties on deforestation

Using both remotely-sensed forest cover change data and a large-scale household survey representative of the smallholder farmer population in the area, we next investigate how the adoption of modern seed varieties translated into changes in land-use decisions by comparing deforestation outcomes in the 60 treatment villages with those in the 32 control villages. As we also randomly varied the level of subsidy among households within treatment villages, we further compare plot level deforestation outcomes between households that received high versus low levels of subsidy. Last, as we randomly varied the share of households receiving subsidies across treatment villages, we were able to estimate whether the extent of village-level exposure matters (see “Methods” section).

We first estimate village-level deforestation using remotely-sensed forest cover change data from Hansen et al.^37^. We compare treatment villages to control villages, where no subsidies for modern seed varieties were given (the excluded category). Within the group of treatment villages, we further distinguish between villages that were randomly selected to get seeds delivered by truck versus those without such delivery.

**Fig. 3:**
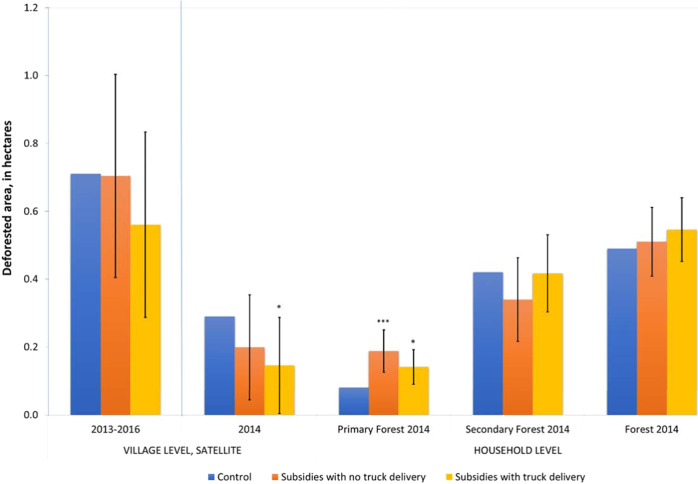
Deforestation measured with village and household survey data. Source: Hansen et al.^37^ and Follow up survey waves 2014. Village-level estimates based on *n* = 92 independent villages included in the experiment; Household-level estimates based on *n* = 904 households living in the *n* = 92 independent villages included in the experiment. Note: Results of village and household level regressions. Dependent variable: deforested area per household, in hectares. For all outcomes, the inverse hyperbolic sine transformation is used (see Supplementary Information, Section A.1). Each bar represents the ordinary least square (OLS) point estimates, and vertical lines show a 90% confidence interval for the difference between treatment and control groups, with standard errors clustered at the village level. Treatment values that are significantly different from the control are indicated with stars. See detailed regression results in Table A.1. Village-level regressions (on the left) use the average of two estimates of village-level deforestation divided by the number of households in the village, the first based on the aggregation of grid cells within the approximate boundaries of the village; and the second based on the aggregation of grid cells within a 5 km radius from the center of the village (these two estimates are shown separately in Table A.11 of the Supplementary Information). The first bar cluster to the left of the blue line shows aggregate deforestation between 2013 and 2016, while the second one (to the right of the blue line) shows deforestation in 2014 to facilitate comparison with household-level data from 2014. Household level regressions on the right show the reported area deforested by the household in hectares in 2014 by forest type (bar clusters 3 and 4) and in total (bar cluster 5). OLS regression, with s.e. clustered at the village level and controlling for a full set of strata fixed effects. *P < 0.10, ***P < 0.01. Source data are provided as a Source Data file (sourcedata.xlsx). The raw data and code used to obtain the estimates are available at https://www.openicpsr.org/openicpsr/project/177141.

The first two bar clusters of Fig. 3 present impact estimates for village-level deforestation, as measured by satellites, showing two separate estimates: the cumulative deforestation at the village level over the period 2013–2016 and that for the year 2014 alone. The year 2014 is the main year when we observe households’ responses to the seed subsidies under study. We also show the estimate for the cumulative deforestation at the village level over the period 2013–2016 because measuring change based on satellite data is more reliable when done over a multiyear period^37^, as this reduces inter-annual measurement error. Moreover, as seeds can be recycled, the cumulative estimate captures the subsequent years during which land use decisions could still potentially be affected by the intervention. For both estimates, we divide the estimated area under deforestation by the number of households in the village. These village-level estimates show no evidence of increases in deforestation over the study period, and, if anything, coefficients are negative. For the 4-year period, the *P*-value of the joint *F*-test = 0.60, the coefficient for lottery without truck = −0.006, the standard error = 0.182, the *P*-value of two-sided *t*-test = 0.972, and the coefficient for a lottery with truck = −0.150, standard error = 0.166, *P*-value of two-sided *t*-test = 0.368. And for the year 2014, the *P*-value of the joint *F*-test = 0.13, while for the lottery without the truck, the coefficient = −0.092, standard error = 0.094, *P*-value of the two-sided *t*-test = 0.337, and for the lottery with a truck the coefficient = −0.144, standard error = 0.086, and *P*-value of two-sided *t*-test = 0.099 (Fig. 3, 1st and 2nd bar clusters and columns 1 and 2 in Table A.1).

The three bar clusters on the right of Fig. 3 present household-level results using measures of deforestation from the household survey data (Fig. 3, 3rd to 5th bar clusters, and corresponding columns 3–5 of Table A.1). The estimates represent the direct effect of being offered a subsidy for the purchase of modern seed varieties on households’ land use decisions in the main agricultural season of 2014. The reported coefficients are interpreted as the effect of the experimental variation on the dependent variable, namely the area of forest cleared for cultivation in hectares by forest type. More precisely, the dependent variable is expressed as the inverse hyperbolic sine transformation of the area in hectares, but at the low average values of per household forest cover, this transformation is approximately equal to the area in hectares. See Bellemare and Casey^38^ and Supplementary Information for an explanation.

The household-level regressions confirm there is no statistically significant impact on overall deforestation (the *P*-value of the joint *F*-test = 0.54). However, when the different types of forest are considered, we find significant impacts on deforestation of primary forest (bar cluster 3, *P*-value of joint *F*-test 0.00), in particular in villages with no truck delivery (coefficient = 0.108, standard error = 0.038, *P*-value of two-sided *t*-test = 0.006). The promotion of modern seed varieties led to a significant increase in deforestation by 0.11 ha per household in villages without truck delivery as compared to the control group. The effect is also significant but lower in villages where seeds were available by trucks (0.06 additional hectares deforested as compared with control villages, standard error = 0.031, *P*-value = 0.052). Because legumes were only delivered by truck, this suggests households with increased relative access to improved cereal seeds decided to clear more fertile land for planting, resulting in a loss of primary forest, while the effect is lower where legumes were also made available. These effects are large: while deforestation of primary forest is much lower than that of secondary forest in the control villages (0.1 hectares compared to 0.4 hectares, in line with more aggregate numbers of ref. ^39^), the relative increase in deforestation of primary forest due to increased access to modern seed varieties represents a more than 100% increase in treatment villages without truck delivery, compared to the mean deforestation rate in the control group. The per-household effect is stronger in those villages where randomly, a larger proportion of households benefited from a subsidy (though not significantly so), possibly due to greater competition for land acquisition as well as collective deforestation (Table A.4 in Supplementary Information).

A limitation of Hansen et al.^37^ data is the absence of differentiation by forest type. We, therefore, test for the robustness of these results using a combination of Hansen et al.^37^ and FACET data (see Methods section). While combining those two datasets adds noise, and the results are not statistically significant, the direction of the results is consistent with the household-level findings. The promotion of modern seed varieties is associated with an increase in the deforestation of primary forest between 2013 and 2016 (stronger in villages without truck delivery) and a decrease in the deforestation of secondary forest. The *P*-value of the joint *F*-test for primary forest is 0.49, with the coefficient for villages without truck delivery = 0.133, standard error = 0.152, *P*-value of two-sided *t*-test = 0.385, while the *P*-value of the joint *F*-test for secondary forest is 0.45, with the coefficient for villages without truck delivery = −0.130, standard error = 0.244, *P*-value of two-sided *t*-test = 0.596. The detailed results are presented in Table A.12.

**Fig. 4:**
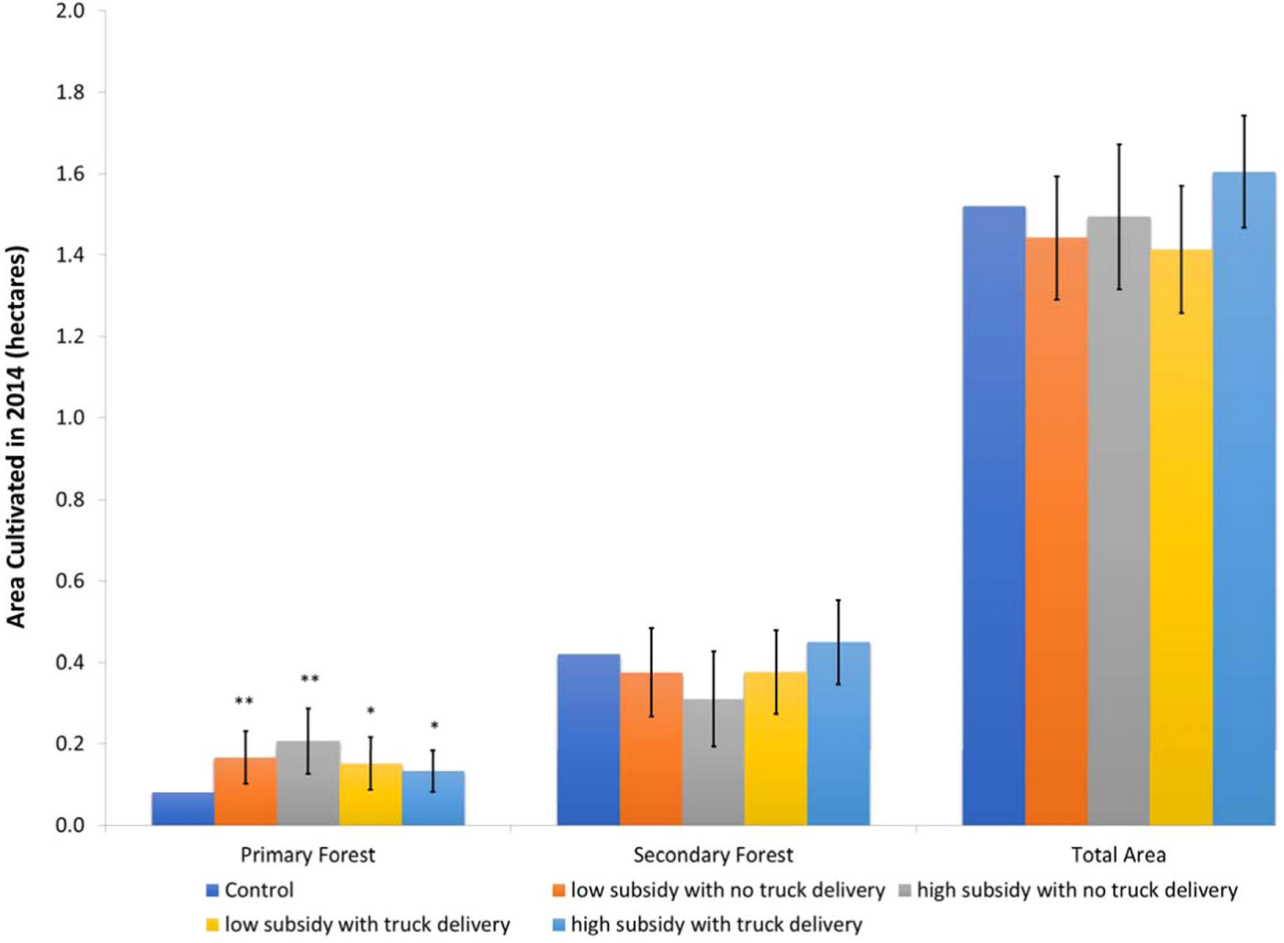
Effect of intervention on area cultivated in 2014 by land use in the previous season, household level. Source: Follow-up survey wave 2014. Estimates based on *n* = 904 households living in the *n* = 92 independent villages included in the experiment. Note: Dependent variable: area cultivated in 2014, in hectares, by type of land use on these plots in the previous season. All outcome variables were measured as the inverse hyperbolic sine transformation of the area in hectares. Each bar represents the ordinary least square (OLS) point estimates, and vertical lines show a 90% confidence interval for the difference between treatment and control groups, with standard errors clustered at the village level. Treatment values that are significantly different from the control are indicated with stars. Based on detailed regressions results in Table A.2. OLS estimation with robust standard errors clustered at the village level and controlling a full set of strata fixed effects. *P < 0.10, **P < 0.05. Source data are provided as a Source Data file (sourcedata.xlsx). The raw data and code used to obtain the estimates are available at https://www.openicpsr.org/openicpsr/project/177141.

Figure 4 shows the effect of promoting modern seed varieties on households’ cultivated area from different land types, distinguishing between households who received high subsidies (90% and 100%) versus low subsidies (30% and 60%), with and without truck delivery (see Table A.2 in Supplementary Information for treatment effects on all types of land). While neither households with low nor high subsidies increased their total farmed land in 2014 (bar cluster 3 and column 7 in Table A.2), they all adjusted the type of land used for cultivation. In particular, we confirm a large percentage increase in acreage of agricultural land originating from primary forest for both low and high subsidy levels (bar cluster 1 and column 2), in line with Fig. 3. The *P*-value of the joint *F*-test for the primary forest is 0.00, with the coefficient for high subsidy in villages without truck delivery = 0.126, standard error = 0.049, *P*-value of two-sided *t*-test = 0.012; the coefficient for low subsidy in villages without truck delivery = 0.086, standard error = 0.039, *P*-value of two-sided *t*-test = 0.028, the coefficient for high subsidy in villages with truck delivery = 0.071, standard error = 0.039, *P*-value of two-sided *t*-test = 0.074 and the coefficient for low subsidy in villages with truck delivery = 0.053, standard error = 0.031, *P*-value of two-sided *t*-test = 0.090. At the same time, there is a reduction in reliance on the secondary forest (bar cluster 2 and column 3), but also of land already under cultivation or fallow (columns 6 and 4 in Table A.2.) The point estimates of deforestation in the primary forest are the largest for high-subsidy households in villages without seed delivery by truck (0.126). These households are also those with the largest point-estimate decline in deforestation of secondary forest (−0.110, with standard error = 0.071, and *P*-value of two-sided test = 0.124). The high subsidy non-truck delivery households deforested an extra 0.126 ha of primary forest on average, while those with a low-level subsidy cleared an extra 0.086 ha of primary forest.

Given the risk of error in plot area measurement, we also estimate the results using a binary dependent variable (i.e., indicating whether a certain type of land was used by the household for cultivation in 2014). These estimates provide a useful robustness test given the relatively high share of households who do not use any land converted from primary forest, which leads to smaller variances of the treatment effect for this variable than for secondary or total forest area. Results are presented in Table A.5 and confirm the overall findings. Results are further robust to using (non-transformed) land size in hectares as the dependent variable, see Table A.6. Overall, the results hence show that households whose high subsidies allowed them to access more seeds of crops with high soil-fertility demands are also the households most likely to have converted the land from the primary forest (while they use less secondary forest).

### Impact of access to improved varieties on labor allocated to field preparation

As labor requirements for land clearing and preparation vary between different land types, land conversion decisions should be reflected in labor allocation. Assessing the impact of modern seed varieties on labor allocation, therefore, helps further address potential concerns regarding measurement errors associated with household-level plot estimates.

More specifically, in the region, clearing primary forest takes more effort than clearing secondary forest or fallow as it requires intensive labor use for the felling of trees and removal of roots. Given the relative scarcity of labor during peak demand periods (including land preparation), and limited opportunities for hiring on the local labor market, these additional labor requirements are likely to lead to a reallocation of households’ labor resources. Indeed, the decline in reliance on secondary forest clearing for high-subsidy households in villages without truck delivery points to such reallocation (see Fig. 4). We collected household labor allocation data to analyze this directly (see “Methods” section).

**Fig. 5:**
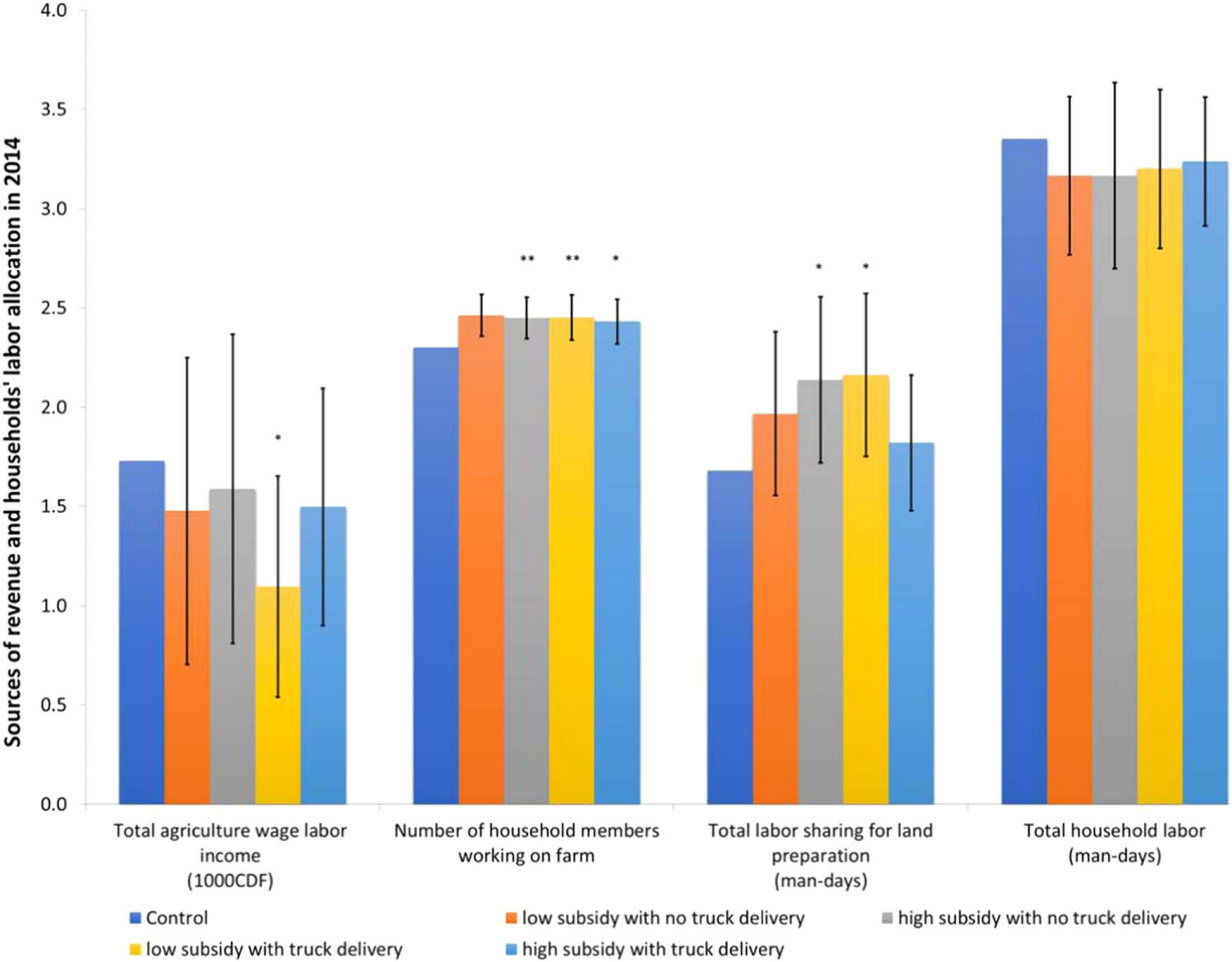
Households’ labor allocation in 2014. Source: Follow-up survey wave 2014. Estimates based on *n* = 839 (bar cluster 1), *n* = 883 (bar cluster 2) and *n* = 902 (bar cluster 3 and 4) households living in the *n* = 92 independent villages included in the experiment. Note: Bar cluster 1 shows the total income earned by household members for agriculture wage labor, bar cluster 2 shows the number of household members working on the household farm, bar cluster 3 is a total number of person-days dedicated to land preparation for this household through labor-sharing arrangements, bar cluster 4 is the number of person-days for land preparation by household members. For all outcomes, the inverse hyperbolic sine transformation is used (see Supplementary Information). Each bar represents the ordinary least square OLS point estimates, and vertical lines show a 90% confidence interval for the difference between treatment and control groups, with standard errors clustered at the village level. Treatment values that are significantly different from the control are indicated with stars. Based on detailed regressions results in Table A.3. OLS estimations with robust standard errors clustered at the village level and controlling for a full set of strata fixed effects. *P < 0.10, **P < 0.05. Source data are provided as a Source Data file (sourcedata.xlsx). The raw data and code used to obtain the estimates are available at https://www.openicpsr.org/openicpsr/project/177141.

While total household labor is unaffected, we find that households in treatment villages allocated more household members to work on the household farm than those in control villages (Fig. 5, 2nd bar cluster, *P*-value for a test of joint significance: 0.00; see also Table A.3 in Supplementary Information). We also find an increase in person days for shared labor between households for land preparation (bar cluster 3, *P*-value for test of joint significance: 0.06). This indicates a shift in labor allocation. Labor sharing is a common practice associated with forest clearing. Villagers organize themselves to collectively clear the forest on each of the group members’ parcels. Qualitative interviews suggest two primary reasons for collective forest clearing. First, it allows the demarcation of the plots to be observed by all members of the group, which is important to establish land rights. Second, the group helps to encourage the motivation of members to complete a cumbersome task.

Consistent with the increased allocation of labor to households farms and labor sharing, we find a decrease in household members’ agricultural wage income (i.e., from wage labor outside the household farm) in the treatment groups, though it is not significant (*P*-value for a test of joint significance 0.25; first bar cluster of Fig. 5). A likely interpretation is that the promotion of modern seed varieties increased the economic returns of family labor for forest clearing, and therefore led to a reallocation of family labor toward this task. However, given the relative isolation of the labor market, there is not enough labor available to meet the increased demand for agricultural expansion and land clearing. This could explain why there is no overall increase in deforestation at the village level (see Fig. 3). Hence the labor data broadly support the overall findings. Because the data does not cover large commercial farms or plantations, we are, however, not able to assess the broader implications for the agricultural labor market and for land use in the commercial sector.

## Discussion

Improved agricultural technologies can make large contributions to economic growth, poverty reduction, and improved health and nutrition in many of the poorest countries in the world. Whether such potential gains come at the cost of environmental sustainability is a long-standing question. The Borlaug hypothesis states that the availability of Green Revolution agricultural technologies (essentially modern seed varieties and fertilizers) helps prevent expanding the land area under cultivation, but theory (such as Jevon’s paradox) and characteristics of local markets, tell us this does not necessarily hold locally.

This question is of particular relevance to DR Congo, home to the second-largest rainforest in the world. Since 2000, deforestation in the Congo Basin has increased substantially, 90% of which is linked to small-scale non-mechanized forest clearing for smallholder agriculture^40^—although artisanal and large-scale commercial operations for logging, mining, and plantations are also shown to have a growing effect on deforestation^41^. Given the high species richness in the remaining primary forest, the expansion of cropland in this region is particularly damaging to biodiversity^42,43^, and policies aimed at agricultural intensification in DRC are often motivated by their potential to reduce pressure on the forest. This justification is used despite the fact that modeling-based analysis warns that such policies, by increasing land rents, may well increase incentives for deforestation^44,45^. Phelps et al.^44^ specifically highlight the need for farm and household-level analyses to better depict the complex realities of on-the-ground land use changes.

The contributions of this paper are twofold: first, we provide a causal test of the impact of the promotion of modern seed varieties on deforestation through a randomized control trial. Second, we establish the importance of distinguishing between primary and secondary forest deforestation and point to the importance of soil fertility considerations.

Our results show that overall, the promotion of modern seed varieties did not lead to an increase in the demand for land or related deforestation, but neither did it reduce land in agricultural use. Hence when considering all deforestation together, we find no strong support for either the Borlaug hypothesis or Jevon’s paradox. Inelasticities in labor supply may explain why there was no change in overall deforestation in the study area. As deforestation is a labor-intensive activity, and opportunities to hire additional labor are limited, households mostly need to rely on the reallocation of a fixed labor supply.

We further find that exposure to modern varieties changed the nature of deforestation. We hypothesized this is driven by an increase in demand for soil nutrients generated by modern seed varieties for cereals, which was met neither by local fertilizer markets nor the government’s agricultural rehabilitation project. In the Congo Basin forest of Equateur, the absence of fertilizers and the lack of information on soil conservation practices meant that previously uncultivated land gave the highest cereal yields—a feature that local farmers are well aware of, following land-and-crop rotation recommendations introduced by agronomists during colonial times. In this setting, smallholder farmers with increased access to modern cereal varieties accelerated the clearing of the primary forest while cultivating less land converted from the secondary forest or other land uses. Exogenous variation in the type of seeds that were offered to farmers helps understand the mechanism. Where cereals were the predominant modern seed variety offered, subsidies led to an increase in the deforestation of the primary forest by beneficiary households. In contrast, modern seed variety promotion led to more limited deforestation in villages where more legumes were available for purchase, in line with the historical recommendations that legumes be planted on poorer soils given their lower need for nitrogen. This last finding comes with a caveat, as the random variation in the availability of cereals versus legumes was not planned for in the original experimental design. The increased access to legumes is found in villages where a larger proportion of treated households used their subsidies and they bought smaller quantities. We cannot fully rule out that these other differences also influenced land conversion decisions. Distinguishing farmers’ soil fertility management decisions for legumes versus cereals hence deserves further research with experimental variation in exposure to different crops. Even so, the results in this study point to the importance of promoting modern seed varieties in combination with soil fertility management practices to achieve sustainable intensification, especially in contexts where smallholders use the primary forest as a source of nitrogen for improved cereals cultivation, as^23^ also concludes.

An important contribution of the study is to demonstrate the value of distinguishing between primary and secondary forest deforestation. The distinction both reflects decision-making at the household level and has major ramifications for biodiversity. Tropical forests support at least two-thirds of the world’s biodiversity^13^ on less than 10% of Earth’s land surface^46^. Primary forest, in particular, is critical for maintaining much of the world’s biodiversity^47–49^. Species abundance and uniqueness are often much lower in secondary than in primary forest^50,51^. And while ecosystem service provision and ecosystem recovery in secondary forest is an active area of research^52,53^, it is well recognized that primary forest provide specific habitat types and characteristics that are not present in secondary forest. Many species are dependent on these unique properties and cannot survive without it^54^. Hence deforestation of primary forest leads to irreversible losses in biodiversity.

Distinguishing between deforestation from primary and secondary forest hence is key to understanding the environmental trade-offs involved with agricultural intensification practices. While modern crop varieties have a large potential to improve livelihoods, the tension with possible negative effects on environmental outcomes needs to be considered in nuanced and context-specific ways. Our results show that improving access to modern cereal varieties in the Congo Basin, in the absence of accompanying policies to improve soil fertility management, may have a high cost for biodiversity due to its effects on the primary forest.

The distinction between forest types can also be important for understanding the carbon sequestration implications of deforestation. While secondary forest can sequester atmospheric CO_2_ 10–20 times faster than primary forest^55,56^, there is also large heterogeneity in carbon sequestration in secondary forest, as it varies with the age of the secondary forest, land-use history, number of times cleared, soil fertility, forest type, fragmentation, etc.^57^ In specific instances, average carbon in the secondary forest can be much lower than in primary forest^32^. Overall, the trade-offs in terms of carbon sequestration deserve further research.

A possible caveat to this study is that it relies on farmers’ ability to distinguish between primary and secondary forest, and imperfect recall may introduce some measurement errors. That said, given the important implications of deforestation of primary forest in terms of labor efforts, it seems reasonable to assume farmers remember the type of land they cleared. In addition, misclassification would bias the results only if it is systematically related to the randomized allocation of treatment, which is unlikely. Another potential caveat is that we rely on farmers’ estimates of plot sizes and, therefore, of the area that was converted from the forest. While these estimates are likely to be imprecise, the main results hold when we consider the extensive margin.

Finally, as is the case for almost any other RCT analyzing agricultural decision-making, a more general caveat is that the interpretation of the results needs to account for the specific context and time period in which they were obtained. The findings are likely most relevant for remote rural areas, with missing or imperfect farm input and output markets and increasing pressure on forest resources. Of course, these are also exactly the types of contexts where deforestation of remaining primary forest is policy relevant. The results contribute to discussions on sustainable intensification practices that aim to reduce productivity-environment trade-offs by stepping away from the modern seed variety plus mineral fertilizer intensification packages^58^ toward context-specific answers, including organic fertilizer and alternative soil fertility management practices^59^. That could include the promotion of legume seeds, associated with less negative consequences for primary forest (see also refs. ^60,61^). If policymakers seek the joint objective of increasing productivity and preserving the primary forest, increased access to modern seed varieties should either be combined with the promotion of sustainable and appropriate technologies and practices to maintain soil fertility or focus on those crops that are less demanding of nitrogen-rich soils.

## Methods

### Context and Intervention

Our study takes place in the former Equateur province, as defined before the 2015 administrative reform. Specifically, the study area covers Nord Ubangi, Sud-Ubangi, and Mongala, which were part of the Equateur province in 2015 and are now defined as three separate provinces. We refer to the Equateur province in the rest of the article, corresponding to the denomination at the time of data collection.

The Equateur province is a remote forested region, part of the broader Congo Basin forest, with high levels of food insecurity and poverty. Households in the province derive most of their income from small-scale subsistence agriculture through shifting cultivation of staples, complemented by the gathering of forest products, fishing, and hunting. The agricultural potential of the region is believed to be large, but productivity is very low, partly due to remoteness and lack of access to modern technologies. Smallholders generally use a limited range of inputs, including handheld tools and low-yielding local seed varieties. The availability of chemical fertilizers is very limited in the region, as are other soil fertility management practices other than the fallowing. And as the agro-climatological conditions are not conducive for large livestock (disease-prone humid forest environment), manure use is limited. Households rely mostly on family labor, and labor availability is constrained in periods of peak labor demand (preparation, planting, and harvesting). Commercialization is hampered by very low road density, poor road quality, and long distances to markets.

The context is further characterized by relative land abundance and the informality of the tenure system. When there is still primary forest within the village boundaries, access to it is allocated by village leaders, clan leaders, or family chiefs, depending on the prevailing allocation system. Once a household has cleared a primary forest plot, a long-term right to cultivate that plot is generally established. That said, there is considerable heterogeneity in the institutional structure of land allocation across villages as a result of inconsistent local agricultural policies, often dating back to the colonial regime.

Farmers practice slash-and-burn cultivation and, therefore, regularly open new fields in the forest. This process requires labor-intensive land preparation. First, trees and woody plants are cut down, and the vegetation is left to dry. Then, before the beginning of the rainy season, the biomass is burnt to create a layer of nutrient-rich ash and eliminate pests and weeds. Farmers typically cultivate several plots during the main agricultural season (average plot size: 0.6 hectares, average number of plots: 4.4). Some of those plots are on land that was already under cultivation the year before, while others require clearing of primary or secondary forest or overgrown fallow land. Household decisions regarding which fields to cultivate take place during the dry season preceding the main rainy season. Qualitative interviews conducted as part of the study’s fieldwork revealed that farmers are keenly aware of the soil fertility implications of those decisions. There is also a short agricultural season from August to November, during which only part of the farmers cultivate, but as it is uncommon to open new fields in the forest for the short agricultural season, we concentrate on the main season.

The main staples grown in the region are maize, rice, groundnuts, cassava, soya, cowpea, yam, and plantain. The common cropping pattern (with local variations) is rotation starting with cereals in land cleared in the forest or overgrown fallow. This is generally followed by groundnuts or other legumes and then cassava for about 2 years, after which land is reverted to fallow for several years (typically for a minimum of two and up to ten years). This pattern aligns with historical agronomic recommendations in the region, according to which freshly deforested land should first be used for cereals, given their high nitrogen requirements. When soils are nitrogen-depleted, legumes can be planted as they fix nitrogen from the atmosphere. While land preparation is generally entrusted to men, most of the agricultural activities until harvest (such as weeding) are entrusted to women or done jointly. Women are also in charge of the cassava plots, where they collect the quantities necessary for the preparation of the main meal on a daily basis.

In this context, the Agricultural Rehabilitation and Recovery Support Project (PARRSA) was implemented starting in 2011, as the main policy tool used by the DRC’s Ministry of Agriculture, with support from the World Bank, to help intensify Equateur’s agricultural sector at the time. An important part of the project focused on developing a market for modern seed varieties, including high-yielding varieties of maize and groundnuts, short-cycle rice, and a disease-tolerant variety of cassava. Promotion of modern seed varieties included: assistance to the national agronomic research institute to resume production of modern seed varieties; subsidies for seed multiplication by local seed multipliers; support to local agricultural extension agents to set up village-level demonstration plots; and a one-time farmer-level seed subsidy intervention that took place in February 2013 and which is the focus of this study. To address low productivity, the varieties that were promoted were selected by the project staff from the ministry of agriculture in partnership with the leading agricultural research agency (INERA) and the national seed service (SENASEM) for their productivity gain potential. Importantly, the government’s intervention only targeted production and access to seeds, and the issue of soil fertility was not addressed. It is not uncommon for agricultural development programs in parts of Sub-Saharan Africa to provide subsidies to facilitate crop varietal change, even if other countries in the region have invested heavily in mineral fertilizer subsidies. The PARRSA project was later restructured and received additional financing, partly to better incorporate land use management and deforestation concerns.

### Experimental design

We use a randomized control trial (RCT) to examine the relationship between the adoption of modern seed varieties and land use decisions by smallholder farmers. The RCT evaluated the PARRSA program described above in a sample of 92 villages spread across Sud Ubangi, Mongala, and North Ubangi districts of the former Equateur province and across 5 (out of 9) territories in those districts, selected based on their relative accessibility by truck.

The sample of villages was stratified based on population size (below or above the median size), remoteness (an indicator of accessibility), and exposure to agricultural extension. Based on this stratification, 32 villages were randomly selected as control villages, and 60 were randomly selected for a pilot subsidy intervention promoting maize, rice, and groundnuts seeds—this paper does not comment on the adoption of cassava, as it was not widely available through the project and is less demanding in terms of soil nutrients.

In the 60 treatment villages, households were randomly chosen ahead of the agricultural season to receive discount vouchers for the purchase of modern seed varieties at a subsidized price through a public lottery organized by the PARRSA team. The vouchers could be used for any of four of the main local staple crops and were redeemable (within 3 months) at the stores of local seed multipliers. The level of subsidy offered by the vouchers was randomly varied across recipients to study households’ decisions to purchase modern seed varieties at different effective prices. Seed vouchers offered either a 30%, 60%, 90%, or 100% subsidy for a maximum of 10 kg of cereal or legumes seeds (or equivalent amounts of cassava stems). This provides the exogenous variation needed to analyze the impact of the promotion of modern seed varieties on household land use decisions. In each treatment village, equal shares of vouchers offering each level of subsidy were distributed. Half of the vouchers were randomly assigned to the household heads, and the other half were assigned to the spouse of the household head. In the case of polygamous households, randomization was conducted to determine which spouse would receive the voucher.

To assess potential spillovers and general equilibrium effects, the proportion of households receiving a voucher was also varied randomly across villages. In one-third of randomly selected villages, 70% of households received a voucher through this lottery (high density); in one-third of random villages, 45% of households received vouchers (medium density), and in the last one-third of villages, only 20% received vouchers (low density). To maximize power, we pooled together villages with different treatment densities (i.e., with different shares of households that received vouchers). Table A.4 shows the results separately.

Last, as the stores of local seed multipliers tend to be located in distant urban areas, another experimental variation was introduced to assess the influence of high transportation costs. In 35 randomly selected villages, a truck offered the seeds for sale directly in the center of the village a few days after the voucher distribution. This allowed households to redeem their voucher and obtain seeds directly in their own village, saving on travel costs. Vouchers were, in theory, redeemable for cereals, legumes, or cassava. Due to logistical considerations, however, trucks ended up offering both legumes and cereals for sale in the villages, while very limited quantities of legumes were available in the stores. In fact, the Ministry ensured the supply of groundnuts for the trucks, while groundnuts ended up in short supply at the seed multipliers’ offices due to mass buying by other institutional buyers for emergency food aid for the Central African Republic. Although unintended, this variation allows insights to be drawn into the differential impact of the use of cereal versus legume modern seed varieties.

In total, 4394 vouchers were distributed across the 60 villages. Random distribution of vouchers in treatment villages was conducted in February 2013. For all varieties, seeds can be re-used in the following seasons (with seeds retaining their improved properties for several seasons). It is important to note that the timing of the distribution of the vouchers in February 2013 was such that it was too late to clear the primary forest that year. In contrast, for the first season of 2014, farmers had time to optimally choose and prepare the plot on which to grow the seeds resulting from the first harvest of these improved seeds. For this reason, we concentrate the analysis on 2014. Survey data confirm farmers re-used the improved seeds and largely did not share them with other farmers.

Given the large number of vouchers distributed, and the household level randomization, the study design provides the statistical power to detect the direct impacts of receiving vouchers and differences between different levels of subsidies. The randomized assignment of treatments across villages (with and without truck delivery and control) and across households in treatment villages (different levels of discount vouchers) assures that villages and households exposed to different treatments are similar in expectation. Results are, therefore, by construction unlikely to suffer from bias due to factors such as those that would arise if obtaining a voucher was correlated with the crops that farmers were growing at baseline or with differentiated market access across villages. Even so, with 92 villages and 3 treatment groups, the design is still somewhat vulnerable to potential accidental imbalances. We therefore also show results based on randomization inference, which shows the probability that a similar size treatment effect would have been observed under different hypothetical realizations of the chosen randomization method. The randomization inference results confirm all main findings. For more detailed information on the experimental design, compliance and take-up, and related data collection, see^36^ and Supplementary Methods.

### Data

In the 92 villages, the study combines remote sensing-based, household, and plot-level survey data to measure forest conversion for agricultural purposes.

### Remote sensing data

At the village level, we use estimates of village-level deforestation derived from Hansen et al.^37^, a global dataset that provides estimates of forest cover in 2000, forest cover loss between 2001 and 2016, and an estimate of the year of forest loss at a 30 square meter resolution. We defined forests as areas with a tree cover taller than five meters and a canopy cover of more than 90 percent in 2000. We define a grid cell as being deforested if it had a canopy tree cover of more than 90% in 2000 and went to a tree cover of less than 25% in a given year. In other words, we restricted the analysis to grid cells with more than 90% tree cover in 2000—similar results are obtained when using an 80% threshold. This definition is more restrictive than the definition used by^37^ (which uses 25% of canopy tree cover) in order to focus on areas of dense forest.

One limitation of the^37^ dataset is that it does not differentiate between types of tree cover, and therefore does not allow to differentiate between deforestation of primary forest, mature secondary forest, or another secondary forest, as done by Tyukavina et al^40^ for the Congo Basin or Hedges et al.^62^ for Haiti for example. FACET (Forêts d’Afrique Centrale Evaluées par Télédétection) atlases, on the other hand, distinguish primary and secondary forest (http://carpe.umd.edu/carpemaps/) using Landsat EMT and data re-sampled at 60 m resolution. For FACET, a primary forest is defined as a mature forest with greater than 60 percent canopy cover, including old-growth forests, plantations, mature trees, and forest galleries, with a mature, heterogeneous canopy height and structure that extinguishes incoming light, reduces reflectance and makes these forests appear dark in satellite imagery. Secondary forest are defined as including recently regrown forests following disturbances, also with greater than 60 percent canopy cover but characterized by uniform canopies that increase reflectance, particularly in the near-infrared wavelengths, when compared to primary forest. The deforestation estimates from FACET are available for 5-year intervals until 2010 and at a lower (60 m) resolution. As FACET is not available for the year(s) following the RCT, we show results based on a combination of^37^ and FACET datasets in Table A.12 in the Supplementary Information.

**Fig. 6:**
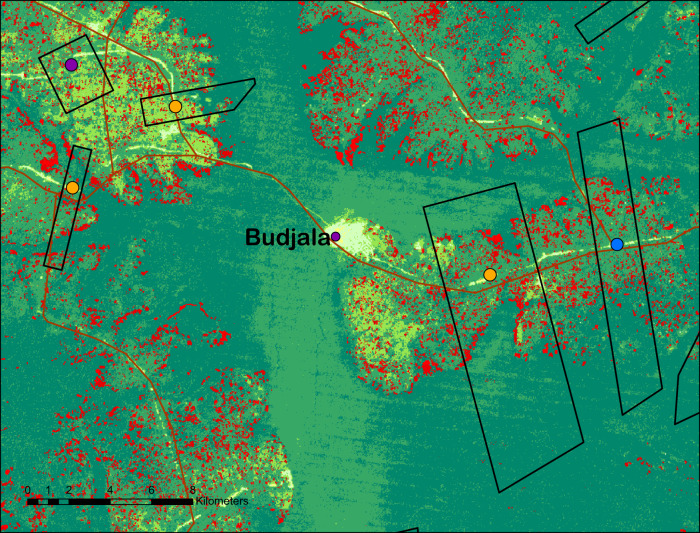
Map of forest cover and forest cover loss at the village level. Note: Close-up of an example of village shapes based on village surveys. The dark green areas are those with a canopy tree cover greater than 90%. The cells marked in red are those deforested between 2013 and 2016, as per the Hansen dataset.

For all the remote sensing-based analyses, the village-level deforestation outcome is calculated as follows: for each village, we sum the grid cells within the village boundaries that were deforested between 2013 and 2016, weighted by the canopy tree cover of the cell in 2000. Official maps of village boundaries, however, do not exist, and the boundaries are difficult to access and often subject to disputes with neighboring villages. In the absence of village shapefiles, we use two estimates of village-level deforestation. The first is based on the aggregation of grid cells within the approximate boundaries of the village. To draw approximate village boundaries, we registered the geo-coordinates of the beginning and the end of the village on all the roads crossing the villages (which are easy to identify and are not likely to be a subject of dispute), and asked a group of community informants to estimate the approximate distances to the village boundaries in every direction. We then use this information and georeferenced road network data to draw approximate village shapes. Figure 6 shows the estimated shapes for some control and treatment villages, as well as their forest cover in 2000 and deforestation between 2013 and 2016. The second approach is based on the aggregation of grid cells within a 5 km buffer from the center of the village. These two estimates are shown separately in Table A.11. Because both of these strategies imply measurement error, the deforestation index (dependent variable in the village-level regressions described below) is calculated as the average of both these outcomes. Results are robust to using the estimates for each method as the main outcome (see Table A.11) and to using a buffer with a radius of 10 km.

Last, to facilitate comparison with the data from the household survey described below, the deforested area is divided by the total population of the village.

### Household survey data

To obtain much more granular information on the relationship between program exposure and land conversion, we conducted a household survey through which we obtained farmers’ own classification of previous land cover and forest categories for each of the plots cultivated in 2014. For land converted from the forest, respondents were asked to distinguish between primary forest, which has no observable traces of previous cultivation, and secondary forest, which is a more recent regrowth on land that had been previously cultivated. As explained above, farmers are strongly aware of this distinction, both because the first clearing often implies subsequent property rights and given that labor efforts for clearing the more dense primary forest are substantially larger. As a result, farmers’ categorization of primary versus secondary forest maps closely into common definitions and is notably consistent with the definition of The Food and Agriculture Organization, which defines primary forest as “naturally regenerated forests of native tree species, where there are no clearly visible indications of human activities and the ecological processes are not significantly disturbed”^30^. Accordingly, secondary forest are defined as “forests regenerating, largely through natural processes, after a significant disturbance of the original forests and displaying major differences in forest structure and/or species composition compared to pristine primary forest.”^29^. Our survey data further shows that the average period of regrowth (i.e., the period between the last crop cultivation and the new forest clearing) for forests that farmers regard as secondary forest are 20 years. Even though farmers may, in some cases, include primary forest land that has been cultivated in a much more distant past, it is clear that what they refer to as primary forest is older growth likely to be of higher ecological value. While more precise remote sensing-based measures of biodiversity are under development^63^, primary forest cover is a convenient proxy for biodiversity.

Plot-level data on conversion decisions come from a household survey collected 1.5 years after the voucher distribution (July 2014), after planting for the main season was completed. A second wave of data was collected after the harvest between November 2014 and March 2015. This is the main source of plot-level data used in the analysis, as its timing allows us to fully capture the impact of modern seed varieties on households’ conversion decisions. The household head (often a man in this context) was asked for detailed information about all the plots the household cultivated in the main season of 2014, including crop use, prior use of the plot, the period of fallow, planting and harvesting dates, tenure, etc. A separate module asked for recall data, for each plot, on agricultural labor for all main agricultural activities (clearing, land preparation, sowing, weeding, and harvesting), separately for men and women in the household, estimating both the number of people and the hours worked to obtain an estimate of total man-days. The module similarly asked for any hired labor and participation in labor exchanges. Data on agricultural wage labor income by household members come from an income module with all household members. Household surveys are further described in the Supplementary Methods and Supplementary notes. Quantitative data collection and questionnaire design was preceded by extensive qualitative interviews and piloting before each wave. Qualitative fieldwork was also used to deepen understanding of farmers’ land conversion decisions and to triangulate findings.

Sample selection for the surveys was based on village census and administrative data from the voucher distribution. In each of the treatment villages, we randomly drew two beneficiaries assigned the 30, 60, and 90% subsidy and three beneficiaries assigned the 100% subsidy for a total of 9 households per treatment village. The sample selection was stratified based on the households’ membership in producer organizations, on having a leadership position in the village, and on polygyny. In each control village, 12 households were randomly selected based on the same stratification criteria.

From the household data, we can exploit differences in subsidy levels at the household level to measure forest conversion decisions at a granular (plot)-level and to distinguish between primary and secondary forest. We further rely on households’ labor allocation for field preparation as an indirect measure of deforestation. Given the limited supply of labor at peak demand times in the study area and the labor intensity of tree cover removal, changes in land use decisions should translate into labor reallocation at the household level.

### Estimation

We estimate the intent-to-treat (ITT) impacts of receiving a price subsidy for improved seeds on both village-level deforestation outcomes and household-level variables capturing households’ decisions on the conversion of primary and secondary forest and related land and labor allocations. Our empirical approach for the household level estimations is summarized in the equation below:

1 $${y}_{h}=\alpha+{{{{{{{{\bf{T}}}}}}}}}_{h}^{{\prime} }\beta+{{{{{{{{\bf{Z}}}}}}}}}_{h}^{{\prime} }\lambda+{\epsilon }_{h}$$

where *y*_*h*_ measures the household’s use of diverse types of land (labor) for the 2014 season, *T*_*h*_ is a vector of variables encompassing the experimental variations described previously and their interactions, and *Z*_*h*_ is a vector of variables including all stratification variables. *ϵ*_*h*_ is a mean zero error term, corrected for village-level clustering. We test for the robustness of the inference, using randomization inference and a Wald omnibus test accounting for the multiple experimental variations, as well as for the joint significance of the different coefficients within and across equations^64^—see Supplementary information. Village-level estimates follow a similar approach at the village level. All data analysis was done with Stata 14.

### Ethics declaration

The research received IRB approval from the ethics committee of the Paris School of Economics—The Abdul Latif Jameel Poverty Action Lab Europe (reference number CE/2013-004). Informed consent was obtained from all human research participants. Outreach activities to share results from this study were organized with both national and local authorities in the region of study.

### Reporting summary

Further information on research design is available in the Nature Portfolio Reporting Summary linked to this article.

## Supplementary information

Supplementary Information

Reporting Summary

## Data Availability

The authors have permission to use all datasets accessed for this study. Source data are provided in this paper. The data used in this paper are available at: https://www.openicpsr.org/openicpsr/project/177141. This includes all household and remote sensing data. FACET remote sensing data can also be accessed at https://carpe.umd.edu/carpemaps/. For Hansen et al. remote sensing data, see 10.1126/science.1244693. Source data are provided in this paper.

## Source data

Source Data

## Code availability

The code needed to reproduce the results is available at https://www.openicpsr.org/openicpsr/project/177141.
