# Supplementary material for: Impact of small farmers' access to improved seeds and deforestation in DR Congo
Source: Nat Commun. 2023 Mar 23;14:1603. doi: 10.1038/s41467-023-37278-2 (PMC10036623; doi:10.1038/s41467-023-37278-2)
Supplement: Supplementary file 1 — Supplementary Information [file 41467_2023_37278_MOESM1_ESM.pdf]

Impact of small farmers' access to improved seeds and deforestation in DR Congo

Supplementary information

Tanguy Bernard[1], Sylvie Lambert[2], Karen Macours*[2], and Margaux Vinez[3]

[1]Bordeaux School of Economics, Univ. Bordeaux
[2]Paris School of Economics, INRAE
[3]World Bank
*corresponding author: karen.macours@psemail.eu

December 2022

A Supplementary information

A.1 Supplementary Tables

- Detailed Regression Results
- Impact of Treatment Density
- Extensive Margin Results
- Linear Measure of Deforestation
- Interaction with extension services

A.2 Supplementary Methods

- Household survey
- Deforestation Using Remote Sensing Data
- Randomization Inference

A.3 Supplementary Notes

A.4 Supplementary References

A.1 Supplementary tables

Detailed Regression Results

Table A.1 – Deforestation at village and household level

	Village Level, Satellite		Household Level		
	(1)	(2)	(3)	(4)	(5)
	2013-2016	2014	Forest	Primary Forest	Secondary Forest
Lottery without truck	−0.006	−0.091	0.020	0.108***	−0.080
	(0.182)	(0.094)	(0.075)	(0.038)	(0.062)
	[0.972]	[0.337]	[0.795]	[0.006]	[0.201]
Lottery with truck	−0.150	−0.144*	0.056	0.061*	−0.003
	(0.166)	(0.086)	(0.069)	(0.031)	(0.057)
	[0.368]	[0.099]	[0.420]	[0.052]	[0.955]
Strat Vars	Yes	Yes	Yes	Yes	Yes
Observations	92	92	904	904	904
Mean Control	0.71	0.29	0.49	0.08	0.42
**P-v Test of Joint Significance**	0.60	0.13	0.54	0.00	0.42

* $p < 0.1$, ** $p < 0.05$, *** $p < 0.01$.

Source: [2] and Follow up survey waves 2014

Note: Village level regressions use the average of two estimates of village-level deforestation per household, the first based on the aggregation of grid cells within the approximate boundaries of the village; and the second based on the aggregation of grid cells within a 5 km radius from the center of the village (these two estimates are shown separately in Table A.11). The first column shows aggregate deforested area in hectares between 2013 and 2016, while the second column shows the same for 2014 to facilitate comparison with household level data. Household level regressions show the reported area deforested by the household in hectares in 2014, in total (column 3) and by forest type (columns 4 and 5). For all outcomes the inverse hyperbolic sine transformation is used.

OLS regression, robust standard errors in parentheses, clustered at the village level for household level regressions. P-values in square brackets. All regressions control for a full set of strata fixed effects. Statistical tests are two-sided t-tests. The P-value of the F-test of joint significance of all treatment variables reported in the bottom part of the Table.

Table A.2 – Effect of intervention on area cultivated by type of vegetation in 2014, household level

	(1) Forest	(2) Primary Forest	(3) Secondary Forest	(4) Fallow	(5) Savanna and other	(6) Cultivated	(7) Total Area
**Total Area Farmed in (type of land)**							
Voucher low x no truck	0.032	0.086**	-0.045	-0.104	0.124	-0.117	-0.078
	(0.071)	(0.039)	(0.066)	(0.081)	(0.075)	(0.085)	(0.092)
	[0.657]	[0.028]	[0.497]	[0.200]	[0.100]	[0.175]	[0.398]
Voucher high x no truck	0.009	0.126**	-0.110	-0.046	0.097	-0.030	-0.026
	(0.090)	(0.049)	(0.071)	(0.099)	(0.068)	(0.087)	(0.108)
	[0.918]	[0.012]	[0.124]	[0.642]	[0.162]	[0.732]	[0.813]
Voucher low x truck	0.026	0.071*	-0.044	-0.134*	0.033	-0.078	-0.107
	(0.081)	(0.039)	(0.062)	(0.077)	(0.041)	(0.078)	(0.095)
	[0.751]	[0.074]	[0.478]	[0.085]	[0.426]	[0.316]	[0.263]
Voucher high x truck	0.080	0.053*	0.029	0.020	0.029	0.058	0.084
	(0.070)	(0.031)	(0.063)	(0.072)	(0.048)	(0.076)	(0.083)
	[0.257]	[0.090]	[0.650]	[0.782]	[0.548]	[0.445]	[0.316]
Strat Vars	Yes	Yes	Yes	Yes	Yes	Yes	Yes
Observations	904	904	904	904	904	904	904
Mean Control	0.49	0.08	0.42	0.42	0.10	1.00	1.52
P-v Test of Joint Significance	0.55	0.00	0.41	0.31	0.10	0.52	0.68

* $p < 0.1$, ** $p < 0.05$, *** $p < 0.01$.

Source: Follow up surveys wave 2014

Note: Area cultivated in 2014 by type of vegetation on these plots in the previous season. All outcome variables measured in hectares. For all outcomes the inverse hyperbolic sine transformation is used.

OLS Regression. Robust standard errors clustered at the village level in parentheses. P-values in square brackets. All regressions control for a full set of strata fixed effects. Statistical tests are two-sided t-tests. The P-value of the F-test of joint significance of all treatment variables reported in the bottom part of the Table.

Table A.3 – Sources of revenue and labour sharing for land preparation in 2014

	(1) Total agri culture wage labor income	(2) Number of HH members working on farm	(3) Total Labor Sharing land preparation	(4) Total Labor
Voucher low x no truck	-0.253	0.162**	0.287	-0.185
	(0.470)	(0.064)	(0.251)	(0.203)
	[0.592]	[0.012]	[0.255]	[0.366]
Voucher high x no truck	-0.141	0.149**	0.458*	-0.184
	(0.473)	(0.063)	(0.254)	(0.239)
	[0.767]	[0.021]	[0.074]	[0.443]
Voucher low x truck	-0.634*	0.151**	0.482*	-0.150
	(0.339)	(0.069)	(0.249)	(0.204)
	[0.065]	[0.030]	[0.056]	[0.465]
Voucher high x truck	-0.233	0.131*	0.140	-0.112
	(0.364)	(0.068)	(0.208)	(0.165)
	[0.524]	[0.058]	[0.501]	[0.500]
Strat Vars	Yes	Yes	Yes	Yes
Observations	839	883	902	902
Mean Control	1.73	2.30	1.68	3.35
P-value test low-high without truck	0.87	0.83	0.46	1.00
P-value test low-high with truck	0.31	0.75	0.10	0.80
**P-v Test of Joint Significance**	0.25	0.00	0.06	0.33

* $p < 0.1$, ** $p < 0.05$, *** $p < 0.01$.

Source: Follow up survey wave 2014

Note: Column 1 shows total income earned by household members for agriculture wage labour, column 2 shows the number of household members working on the household farm, column 3 is total number of person-days dedicated to land preparation for this household through labour-sharing arrangements, column 4 is the number of person-days for land preparation by household members. For all outcomes the inverse hyperbolic sine transformation is used (see Supplementary Material).

OLS regression. Robust standard errors clustered at the village level in parentheses. P-values in square brackets. All regressions control for a full set of strata fixed effects. Statistical tests are two-sided t-tests. The P-value of the F-test of joint significance of all treatment variables reported in the bottom part of the Table.

Impact of Treatment Density

Table A.4 shows results separately for each of the treatment density levels.

Extensive Margin Results

Tables A.1 and A.2 use the estimated area deforested per household as the outcome variable. Because the distribution of these variables is characterized by many zero values and a long right hand side tail, and for consistency throughout, we use the inverse hyperbolic sine transformation of the area for both the village level and household level estimates. For similar reasons, Table A.3 also uses the inverse hyperbolic sine transformation for the labour variables, which are measured by the number of person-days used on the plot for land preparation (mostly felling of trees and clearing of land). Table A.5 shows an alternative estimate for Table A.2 with results on the extensive margin: in each column, the dependent variable is binary and the reported coefficients are interpreted as the effect of the experimental variation on the probability that a household decided to cultivate on a particular type of land in the main 2014 season. Results on the extensive margin are clearest for households in villages where no truck delivered the seeds (which also means where groundnuts were likely not available for purchase at offices of seed-multipliers). There, receiving a voucher led to a more than twofold increase in the probability that a household used land from the primary forest (column 2).

Table A.4 – Deforestation at village and household level

	Village Level, Satellite		Household Level		
	(1)	(2)	(3)	(4)	(5)
	2013-2016	2014	Forest	Primary Forest	Secondary Forest
Voucher x no truck x density 20	−0.129	−0.164	−0.087	0.061	−0.154**
	(0.272)	(0.141)	(0.081)	(0.049)	(0.061)
	[0.638]	[0.248]	[0.285]	[0.213]	[0.013]
Voucher x no truck x density 45	−0.222	−0.184	0.100	0.127	−0.010
	(0.271)	(0.140)	(0.124)	(0.081)	(0.092)
	[0.415]	[0.194]	[0.423]	[0.119]	[0.917]
Voucher x no truck x density 70	0.286	0.051	0.031	0.126**	−0.084
	(0.258)	(0.134)	(0.109)	(0.049)	(0.096)
	[0.272]	[0.702]	[0.774]	[0.012]	[0.383]
Voucher x truck x density 20	−0.241	−0.224*	0.072	0.045	0.023
	(0.235)	(0.122)	(0.094)	(0.045)	(0.079)
	[0.308]	[0.070]	[0.447]	[0.316]	[0.770]
Voucher x truck x density 45	−0.160	−0.153	0.031	0.059	−0.023
	(0.241)	(0.125)	(0.091)	(0.040)	(0.079)
	[0.508]	[0.223]	[0.734]	[0.143]	[0.770]
Voucher x truck x density 70	−0.050	−0.056	0.064	0.076	−0.010
	(0.232)	(0.120)	(0.107)	(0.051)	(0.075)
	[0.829]	[0.645]	[0.550]	[0.139]	[0.892]
Strat Vars	Yes	Yes	Yes	Yes	Yes
Observations	92	92	904	904	904
Mean Control	0.71	0.29	0.49	0.08	0.42
P-value test 20-70 without truck	0.22	0.22	0.32	0.33	0.47
P-value test 20-70 with truck	0.50	0.26	0.95	0.62	0.71
**P-v Test of Joint Significance**	0.57	0.12	0.56	0.00	0.40

* $p < 0.1$, ** $p < 0.05$, *** $p < 0.01$.

Source: [2] and Follow up survey waves 2014

Note: Village level regressions use the average of two estimates of village-level deforestation per household, the first based on the aggregation of grid cells within the approximate boundaries of the village; and the second based on the aggregation of grid cells within a 5 km radius from the center of the village. The first column shows aggregate deforestation between 2013 and 2016, while the second column shows deforestation of 2014 to facilitate comparison with household level data. Household level regressions show the reported area deforested by the household in hectares in 2014, in total (column 3) and by forest type (columns 4 and 5). For all outcomes the inverse hyperbolic sine transformation is used.

OLS regression, robust standard errors in parentheses, clustered at the village level in for household level regressions. P-values in square brackets. All regressions control for a full set of strata fixed effects. Statistical tests are two-sided t-tests. The P-value of the F-test of joint significance of all treatment variables reported in the bottom part of the Table.

Table A.5 – Household level effect on type of deforestation of 2014, binary outcomes

	(1) Forest	(2) Primary Forest	(3) Secondary Forest	(4) Fallow	(5) Savanna and other	(6) Cultivated
**One or more plots in (type of land) - dummy**						
Voucher low x no truck	0.078	0.127**	-0.039	-0.054	0.053	-0.071*
	(0.071)	(0.051)	(0.066)	(0.076)	(0.060)	(0.038)
	[0.271]	[0.016]	[0.554]	[0.483]	[0.384]	[0.064]
Voucher high x no truck	0.026	0.115***	-0.064	-0.122	0.008	-0.029
	(0.072)	(0.043)	(0.064)	(0.075)	(0.042)	(0.026)
	[0.718]	[0.008]	[0.316]	[0.106]	[0.857]	[0.266]
Voucher low x truck	0.023	0.073	-0.042	-0.089	0.032	0.015
	(0.070)	(0.046)	(0.057)	(0.070)	(0.045)	(0.021)
	[0.747]	[0.121]	[0.465]	[0.204]	[0.488]	[0.484]
Voucher high x truck	0.082	0.039	0.023	-0.014	-0.016	0.018
	(0.064)	(0.033)	(0.059)	(0.063)	(0.037)	(0.022)
	[0.207]	[0.237]	[0.694]	[0.819]	[0.665]	[0.396]
Strat Vars	Yes	Yes	Yes	Yes	Yes	Yes
Observations	903	903	903	903	903	903
Mean Control	0.50	0.09	0.45	0.56	0.08	0.92
P-v Test of Joint Significance	0.33	0.00	0.52	0.20	0.59	0.37

* $p < 0.1$, ** $p < 0.05$, *** $p < 0.01$.
Source: Follow up surveys wave 2014
Note: Type of vegetation in the previous season for plots cultivated in 2014 (dummy).
OLS Regression. Robust standard errors clustered at the village level in parentheses. P-values in square brackets. All regressions control for a full set of strata fixed effects. Statistical tests are two-sided t-tests. The P-value of the F-test of joint significance of all treatment variables reported in the bottom part of the Table.

Linear Measure of Deforestation

As a further robustness test, and to show that the primary forest result is not sensitive to the inverse hyperbolic sine transformation, Table A.6 replicates the results of Table A.2 without the transformation. The mean in the control now gives the direct estimate of the area under cultivation (in hectares). The order of magnitude of the point estimates and the significance for primary forest estimates are similar to those with the inverse hyperbolic sine transformation, which is as expected given the large number of households without any primary forest conversion (large share of zeros).

Table A.6 – Effect of intervention on area cultivated by type of vegetation in 2014, outcome measured in hectares (non-transformed)

	(1) Forest	(2) Primary Forest	(3) Secondary Forest	(4) Fallow	(5) Savanna and other	(6) Cultivated	(7) Total Area
**Total Area Farmed in (type of land)**							
Voucher low x no truck	0.060	0.108*	-0.048	-0.199	0.163*	-0.264	-0.351
	(0.122)	(0.055)	(0.112)	(0.125)	(0.098)	(0.171)	(0.294)
	[0.625]	[0.051]	[0.668]	[0.114]	[0.100]	[0.126]	[0.235]
Voucher high x no truck	0.027	0.201**	-0.173*	0.063	0.251	0.060	0.173
	(0.139)	(0.087)	(0.098)	(0.214)	(0.175)	(0.257)	(0.437)
	[0.844]	[0.023]	[0.081]	[0.768]	[0.154]	[0.815]	[0.693]
Voucher low x truck	0.052	0.093*	-0.041	-0.236*	0.062	-0.148	-0.322
	(0.124)	(0.053)	(0.101)	(0.125)	(0.058)	(0.182)	(0.303)
	[0.679]	[0.081]	[0.682]	[0.062]	[0.286]	[0.417]	[0.291]
Voucher high x truck	0.088	0.072*	0.016	0.030	0.053	0.124	0.238
	(0.100)	(0.041)	(0.090)	(0.127)	(0.071)	(0.183)	(0.287)
	[0.383]	[0.077]	[0.864]	[0.812]	[0.457]	[0.499]	[0.410]
Strat Vars	Yes	Yes	Yes	Yes	Yes	Yes	Yes
Observations	904	904	904	904	904	904	904
Mean Control	0.69	0.10	0.59	0.58	0.13	1.48	2.80
P-v Test of Joint Significance	0.54	0.00	0.43	0.47	0.07	0.71	0.81

* $p < 0.1$, ** $p < 0.05$, *** $p < 0.01$.

Source: Follow up surveys wave 2014

Note: Area cultivated in 2014 by type of vegetation on these plots in the previous season. All outcome variables measured in hectares. This is the same table as table A.2 except the inverse hyperbolic sine transformation is not used.

OLS Regression. Robust standard errors clustered at the village level in parentheses. P-values in square brackets. All regressions control for a full set of strata fixed effects. Statistical tests are two-sided t-tests. The P-value of the F-test of joint significance of all treatment variables reported in the bottom part of the Table.

Interaction with Extension Services

As expected given stratification, the results on deforestation and labour are robust to including the interaction effects with the orthogonal randomized treatment providing extension services, as demonstrated by tables A.7 to A.9. Almost all of the interaction effects are insignificant, suggesting that the additional information regarding modern seed varieties and related practices did not systematically lead to lower or higher impacts on deforestation or labour allocation. That said, the design was not powered to analyze the interaction effects, so no clear conclusion can be drawn on this last point.

Table A.7 – Deforestation at village and household level, interaction with extension

	Village Level, Satellite		Household Level		
	(1)	(2)	(3)	(4)	(5)
	2013-2016	2014	Forest	Primary Forest	Secondary Forest
Lottery without truck	0.221	0.014	0.064	0.117**	−0.054
	(0.331)	(0.172)	(0.107)	(0.051)	(0.108)
	[0.506]	[0.933]	[0.555]	[0.024]	[0.617]
Lottery without truck x extension	−0.328	−0.155	−0.061	−0.010	−0.038
	(0.397)	(0.206)	(0.146)	(0.071)	(0.133)
	[0.411]	[0.456]	[0.677]	[0.886]	[0.777]
Lottery with truck	−0.055	−0.056	0.009	0.025	−0.009
	(0.286)	(0.148)	(0.123)	(0.048)	(0.107)
	[0.849]	[0.708]	[0.940]	[0.606]	[0.933]
Lottery with truck x extension	−0.146	−0.134	0.071	0.054	0.008
	(0.353)	(0.183)	(0.146)	(0.061)	(0.126)
	[0.680]	[0.466]	[0.630]	[0.374]	[0.948]
Strat Vars	Yes	Yes	Yes	Yes	Yes
Observations	92	92	904	904	904
Mean Control	0.71	0.29	0.49	0.08	0.42
**P-v Test of Joint Significance**	0.41	0.09	0.59	0.01	0.45

* $p < 0.1$, ** $p < 0.05$, *** $p < 0.01$.

Source: [2] and Follow up survey waves 2014

Note: Village level regressions use the average of two estimates of village-level deforestation per household, the first based on the aggregation of grid cells within the approximate boundaries of the village; and the second based on the aggregation of grid cells within a 5 km radius from the center of the village. The first column shows aggregate deforested area measured in hectares between 2013 and 2016, while the second column shows deforestation of 2014 to facilitate comparison with household level data. Household level regressions show the reported area deforested by the household in hectares in 2014, in total (column 3) and by forest type (columns 4 and 5). For all outcomes the inverse hyperbolic sine transformation is used.

OLS regression, robust standard errors in parentheses, clustered at the village level for household level regressions. P-values in square brackets. All regressions control for a full set of strata fixed effects. Statistical tests are two-sided t-tests. The P-value of the F-test of joint significance of all treatment variables reported in the bottom part of the Table.

Table A.8 – Effect of intervention on area cultivated by type of vegetation in 2014, household level, interaction with extension

	(1) Forest	(2) Primary Forest	(3) Secondary Forest	(4) Fallow	(5) Savanna and other	(6) Cultivated	(7) Total Area
**Total Area Farmed in (type of land)**							
Voucher low x no truck	0.121	0.120	-0.000	-0.025	0.186	-0.157	-0.024
	(0.119)	(0.078)	(0.117)	(0.137)	(0.189)	(0.123)	(0.137)
	[0.310]	[0.126]	[0.999]	[0.854]	[0.329]	[0.207]	[0.860]
Voucher low x no truck x extension	-0.125	-0.046	-0.064	-0.114	-0.085	0.059	-0.075
	(0.150)	(0.089)	(0.142)	(0.173)	(0.202)	(0.170)	(0.185)
	[0.408]	[0.609]	[0.656]	[0.509]	[0.674]	[0.728]	[0.687]
Voucher high x no truck	0.018	0.114*	-0.097	-0.098	-0.000	-0.195	-0.170
	(0.118)	(0.066)	(0.120)	(0.121)	(0.087)	(0.125)	(0.162)
	[0.879]	[0.087]	[0.422]	[0.421]	[1.000]	[0.123]	[0.297]
Voucher high x no truck x extension	-0.011	0.019	-0.019	0.068	0.137	0.235	0.204
	(0.167)	(0.092)	(0.148)	(0.181)	(0.124)	(0.170)	(0.215)
	[0.946]	[0.836]	[0.897]	[0.709]	[0.274]	[0.171]	[0.345]
Voucher low x truck	-0.124	0.029	-0.150	0.016	0.051	-0.043	-0.094
	(0.125)	(0.058)	(0.105)	(0.147)	(0.070)	(0.117)	(0.155)
	[0.327]	[0.613]	[0.157]	[0.912]	[0.472]	[0.716]	[0.548]
Voucher low x truck x extension	0.225	0.062	0.159	-0.229	-0.027	-0.051	-0.019
	(0.158)	(0.075)	(0.129)	(0.171)	(0.086)	(0.160)	(0.199)
	[0.158]	[0.410]	[0.221]	[0.184]	[0.757]	[0.749]	[0.923]
Voucher high x truck	0.112	0.021	0.101	0.016	0.007	0.080	0.114
	(0.132)	(0.051)	(0.121)	(0.089)	(0.070)	(0.111)	(0.124)
	[0.396]	[0.678]	[0.408]	[0.857]	[0.918]	[0.474]	[0.364]
Voucher high x truck x extension	-0.051	0.048	-0.111	0.005	0.033	-0.031	-0.044
	(0.154)	(0.063)	(0.141)	(0.133)	(0.094)	(0.151)	(0.167)
	[0.742]	[0.448]	[0.434]	[0.968]	[0.723]	[0.836]	[0.791]
Strat Vars	Yes	Yes	Yes	Yes	Yes	Yes	Yes
Observations	904	904	904	904	904	904	904
Mean Control	0.49	0.08	0.42	0.42	0.10	1.00	1.52
P-v Test of Joint Significance	0.58	0.01	0.46	0.31	0.15	0.77	0.79

* $p < 0.1$, ** $p < 0.05$, *** $p < 0.01$.

Source: Follow up surveys wave 2014

Note: Area cultivated in 2014 by type of vegetation on these plots in the previous season. All outcome variables measured in hectares. For all outcomes the inverse hyperbolic sine transformation is used.

OLS Regression. Robust standard errors clustered at the village level in parentheses. P-values in square brackets. Statistical tests are two-sided t-tests. The P-value of the F-test of joint significance of all treatment variables reported in the bottom part of the Table.

Table A.9 – Sources of revenue and labour sharing for land preparation in 2014, interaction with extension

	(1) Total agri culture wage labor income	(2) Number of HH members working on farm	(3) Total Labor Sharing land preparation	(4) Total Labor
Voucher low x no truck	-0.946	0.221*	0.639	-0.305
	(0.628)	(0.122)	(0.472)	(0.377)
	[0.135]	[0.072]	[0.180]	[0.420]
Voucher low x no truck x extension	0.996	-0.085	-0.503	0.159
	(0.891)	(0.144)	(0.565)	(0.448)
	[0.266]	[0.559]	[0.376]	[0.724]
Voucher high x no truck	-0.030	0.056	1.039***	-0.193
	(0.756)	(0.104)	(0.327)	(0.441)
	[0.968]	[0.591]	[0.002]	[0.662]
Voucher high x no truck x extension	-0.149	0.128	-0.832*	0.004
	(0.965)	(0.130)	(0.467)	(0.520)
	[0.878]	[0.329]	[0.078]	[0.994]
Voucher low x truck	-0.835	0.106	0.109	-0.004
	(0.519)	(0.112)	(0.301)	(0.327)
	[0.111]	[0.347]	[0.717]	[0.990]
Voucher low x truck x extension	0.325	0.069	0.554	-0.221
	(0.694)	(0.142)	(0.458)	(0.416)
	[0.641]	[0.630]	[0.230]	[0.596]
Voucher high x truck	0.209	0.254***	0.138	0.152
	(0.577)	(0.083)	(0.305)	(0.223)
	[0.718]	[0.003]	[0.654]	[0.496]
Voucher high x truck x extension	-0.682	-0.190	-0.007	-0.405
	(0.740)	(0.123)	(0.421)	(0.313)
	[0.359]	[0.125]	[0.986]	[0.200]
Strat Vars	Yes	Yes	Yes	Yes
Observations	839	883	902	902
Mean Control	1.73	2.30	1.68	3.35
P-value test low-high without truck	0.27	0.22	0.40	0.69
P-value test low-high with truck	0.06	0.10	0.93	0.42
**P-v Test of Joint Significance**	0.38	0.04	0.06	0.74

* $p < 0.1$, ** $p < 0.05$, *** $p < 0.01$.

Source: Follow up surveys wave 2014

Note: Column 1 shows total income earned by household members for agriculture wage labour, column 2 shows the number of household members working on the household farm, column 3 is total number of person-days dedicated to land preparation for this household through labour-sharing arrangements, column 4 is the number of person-days for land prepraration by household members. For all outcomes the inverse hyperbolic sine transformation is used.

OLS regression. Robust standard errors clustered at the village level in parentheses. P-values in square brackets. Statistical tests are two-sided t-tests. The P-value of the F-test of joint significance of all treatment variables reported in the bottom part of the Table.

A.2 Supplementary Methods

Experimental design, compliance and take-up

The key features of the experimental design are described in the Methods section of the paper. A set of 92 villages was selected for possible targeting of seed subsidies (through vouchers) based on their relative accessibility by truck. The 92 villages are a subset of villages selected for a larger, orthogonal extension experiment. The 92 villages were stratified based on size (below or above median size), remoteness(a subjective indicator of accessibility), and treatment status of the PARRSA extension intervention, which was itself randomized across 200 villages. Respecting this stratification, 32 villages were randomly selected as control villages for the seed vouchers, and 60 for distribution of seed vouchers. The vouchers provided a subsidy to buy improved seeds from specific seed-multipliers, and involved co-financing from the beneficiaries (cash and/or travel costs, except for subsidies of 100% in villages with a truck delivery).

We draw on the administrative data collected during the voucher distribution, and the records of seed sales (obtained both for sales from the trucks (delivery) and at the seed-multipliers offices). Each voucher indicated the name and identifier of the household that received it, and details about the sale were registered each time a voucher was used (including identity of the buyer, identifier of the voucher, and quantities of seeds bought). There was full compliance with the experimental design: the public lotteries were organized in all 60 randomly selected treatment villages, the 30 villages randomly selected for truck delivery received truck delivery, and none of the 32 randomly selected control villages received vouchers or truck delivery.

The administrative data further shows that vouchers were very successful in convincing households to get seeds, and that voucher take-up is higher for higher subsidy levels. In villages with truck delivery, the rate of use of vouchers was very high. More than 90% used their 100% voucher, and 77% of the 90% voucher recipients used it as well. This figure drops to 56 and 48% for 60 and 30% vouchers respectively. As expected, voucher use is lower in villages where households had to cover travel costs, but remains high. In those villages, close to 46% of households used their 100% vouchers. 40% for 90% vouchers, 22% for 60% vouchers, and 14% for 30% vouchers. There are no big differences in take-up in villages where a higher proportion of households received vouchers. In villages without truck delivery, it is slightly stronger for higher share of households treated: 36% of voucher recipients used their voucher in villages were 70% of households were given one, 37% in villages were 45% received one, and 22% in villages were 20% of households received one. In villages with a truck delivery, around 70% of voucher recipients used their vouchers irrespective of the percentage of households who received one. Overall, these take-up rates suggest demand is highly sensitive to price but there appear not to be strong non-linearities in the demand curves.

The vast majority of sales were for groundnuts, rice and maize seeds. In villages with a truck delivery, among those who redeemed their voucher, 72% bought groundnuts, 37% bought rice, and 28% bought maize. In villages without a truck delivery, 41% bought rice, 25% bought maize, and 23% bought groundnuts. If a relatively large share of households who redeemed a voucher used it to buy soybean in village with no truck delivery (29%), virtually none did so in truck villages. The difference between villages with and without truck delivery is largely supply driven, as groundnuts were not always available at the seed-multipliers offices. In terms of quantities of seeds obtained, households from villages without truck delivery who decided to use their voucher bought an average of 9.6 kilograms of seeds regardless of the level of the subsidy. When a truck delivery took place after the lottery, however, households with lower levels of subsidies (30 and 60%) only bought an average of 5.4 kilograms while households with high subsidies (90 and 100%) bought close to 10 kilograms on average.

Household survey

A first household survey was conducted between November and December 2013, i.e. after the agricultural season directly following the lotteries. Data was collected in the 92 villages involved in the lottery experiment. For the 60 voucher treatment villages, administrative records about the beneficiaries' identity and type of seed voucher received during the lotteries is also available. This administrative data was used for the sample selection of the follow-up surveys. In particular, in each of the voucher villages, we randomly drew two beneficiaries, stratified by level of subsidy and gender (Male/Female; 30%/60%/90%/100%). A third beneficiary household was added for the 100% subsidy level to maximize power. In each village, data was also collected on 3 random households who were not assigned any vouchers.[1] This gives a first group of 12 households, for whom detailed information about agricultural production in the season after voucher distribution, in addition to information on take-up, perceptions and social networks was collected. Given that the voucher distribution was random, these households can be compared to 12 randomly drawn households in the control. In all villages, the samples were further stratified on baseline membership in producer organizations, on having a leadership positions in the village, and on polygyny. For an additional 10 random households (1 men and 1 women for each subsidy level) a short survey was implemented on take up, perceptions and social networks. This sample again includes 2 people with leadership positions.

For the second follow-up household survey in the 92 villages, used to analyze conversion decisions, the same 12 households for whom we had detailed agricultural information in the first follow-up survey have been resurveyed. To increase power, six additional households for whom only a short survey was implemented in 2013 were added to the sample (two each with 0, 90 and 100% subsidies (one man, one woman for each level) in the voucher villages. Finally, an additional 6 household with 0 or 100% reduction and 2 with 90% reduction were added in

[1]These non-beneficiary households are not used in the current analysis.

the truck delivery villages. The household head was asked to draw a map of all plots cultivated by the household, and for each plot we obtained information on crop choice, the plots use in the prior season (primary forest, secondary forests, savannah, fallow, or cultivated), and the estimated plot size. Plot size is measured as reported by the farmer, as independent direct measurement wasn't possible to systematically obtain given the geographical dispersion of plots and particular access difficulties for recently cleared plots in the forest. A separate plot-level module was used to collect detailed information on labour allocation for land preparation and cultivation for each plot, and a different module collected information on household's economic activities. [1] provide more details on data collection instruments.

The unique advantage of the household-plot level data is that it allows for analysis at very fine level of granularity of the type of deforestation different households engaged in (average plot size is 0.6 hectares, average number of plots being 4.4), and, given the randomization, to directly analyze the causal link between whether a household received access to subsidized seeds (and at what price) and its decision to clear primary or secondary forest. It does rely, however, on households' ability to distinguish between primary and secondary forest. Given that the density of primary forest implies substantially larger efforts for clearing, we believe this is a reasonable assumption in this setting. Indeed, the aggregate estimates of deforestation of primary and secondary forests in the control villages are close to those estimated by Potapov et al. [3] for 2010 using FACET (see below). More importantly, it seems unlikely that any misclassification would be systematically related to the randomized intervention, and while it may hence cause some random noise in the outcome variable, it should not systematically bias any of the results.

For the main results in this paper we focus on the sample of households who were surveyed both in 2013 and 2014. As a robustness test, results for the full sample (including households added in 2014) are presented in Table A.10.

Table A.10 – Effect of intervention on area cultivated by type of vegetation in 2014, extended household sample

	(1) Forest	(2) Primary Forest	(3) Secondary Forest	(4) Fallow	(5) Savanna and other	(6) Cultivated	(7) Total Area
**Total Area Farmed in (type of land)**							
Voucher low x no truck	0.030	0.086**	-0.046	-0.098	0.123	-0.113	-0.073
	(0.071)	(0.039)	(0.065)	(0.081)	(0.075)	(0.085)	(0.092)
	[0.671]	[0.031]	[0.478]	[0.230]	[0.103]	[0.188]	[0.428]
Voucher high x no truck	0.037	0.095**	-0.054	-0.063	0.101	-0.034	-0.019
	(0.085)	(0.039)	(0.069)	(0.090)	(0.064)	(0.077)	(0.099)
	[0.665]	[0.018]	[0.436]	[0.489]	[0.120]	[0.659]	[0.846]
Voucher low x truck	0.017	0.068*	-0.051	-0.110	0.030	-0.072	-0.093
	(0.079)	(0.038)	(0.061)	(0.077)	(0.041)	(0.077)	(0.094)
	[0.826]	[0.080]	[0.411]	[0.157]	[0.463]	[0.355]	[0.325]
Voucher high x truck	0.032	0.046	-0.006	0.003	0.038	0.017	0.040
	(0.068)	(0.031)	(0.056)	(0.067)	(0.044)	(0.068)	(0.083)
	[0.644]	[0.139]	[0.911]	[0.969]	[0.396]	[0.801]	[0.635]
Strat Vars	Yes	Yes	Yes	Yes	Yes	Yes	Yes
Observations	1261	1261	1261	1261	1261	1261	1261
Mean Control	0.49	0.08	0.42	0.42	0.10	1.00	1.52
P-v Test of Joint Significance	0.63	0.01	0.45	0.30	0.09	0.42	0.63

Source: Follow up survey wave 2014, full sample

* $p < 0.1$, ** $p < 0.05$, *** $p < 0.01$.

Note: Area cultivated in 2014 by type of vegetation on these plots in the previous season. All outcome variables measured in hectares. For all outcomes the inverse hyperbolic sine transformation is used.

OLS Regression. Robust standard errors clustered at the village level in parentheses. P-values in square brackets. All regressions control for a full set of strata fixed effects. Statistical tests are two-sided t-tests. The P-value of the F-test of joint significance of all treatment variables reported in the bottom part of the Table.

Deforestation using remote sensing data

Table A.11 presents the results for the two different estimates of village level deforestation separately. As explained in the Data section our main results are based on the average of the two estimates. Using approximate village boundaries or a 5 km buffer around the village center instead yields similar results.

Table A.11 – Deforestation using different village shapes

	Circle 5 km		Village Shape	
	(1) 2013-2016	(2) 2014	(3) 2013-2016	(4) 2014
Lottery without truck	-0.006	-0.118	0.043	-0.020
	(0.215)	(0.121)	(0.143)	(0.066)
	[0.979]	[0.331]	[0.763]	[0.766]
Lottery with truck	-0.086	-0.138	-0.218*	-0.118*
	(0.197)	(0.111)	(0.131)	(0.060)
	[0.662]	[0.217]	[0.100]	[0.053]
Strat Vars	Yes	Yes	Yes	Yes
Observations	92	92	92	92
Mean Control	0.79	0.33	0.55	0.22
P-v Test of Joint Significance	0.80	0.20	0.46	0.21

* $p < 0.1$, ** $p < 0.05$, *** $p < 0.01$.

Source: Follow up surveys wave 2014 and [2]

Note: Village level regressions use estimates of village-level deforested area (in hectares) per household, the first based on the aggregation of grid cells within the approximate boundaries of the village; and the second based on the aggregation of grid cells within a 5 km radius from the center of the village. The first column and third columns show aggregate deforestation between 2013 and 2016, while the second and fourth columns show deforestation of 2014 to facilitate comparison with household level data.

OLS regression, robust standard errors in parentheses. P-values in square brackets. All regressions control for a full set of strata fixed effects. For all outcomes the inverse hyperbolic sine transformation is used (see Supplementary Material). Statistical tests are two-sided t-tests. The P-value of the F-test of joint significance of all treatment variables reported in the bottom part of the Table.

To our knowledge, there are no satellite-based data products that would allow differentiating deforestation of primary and secondary forests for the relevant time frame. FACET The Observatoire Satellital des Forets d'Afrique Centrale (FACET, which stands for Monitoring the forests of Central Africa using remotely sensed data sets), provides classifications of forest cover and forest cover loss distinguishing between primary and secondary forests for the periods 2000-2005-2010, but at a lower resolution. Combining it with [2] data on deforestation is nevertheless possible, but implies some noise. To do so, we use FACET for forest cover in 2000 and Hansen for annual forest losses between 2000 and 2012. We first combine the FACET and Hansen rasters using the spatial analyst tool in Arcgis. Because the rasters have different spatial resolutions, the raster with the smaller cell size is automatically resampled using nearest neighbor method to the resolution of the dataset with the coarsest resolution,

and therefore precision is lost. The combined dataset will have, for each cell, information on forest cover in 2000 (from FACET) and on forest losses (from Hansen). Using the combined dataset, we keep only pixels that are classified as primary or secondary forest, and compute deforestation within the village boundaries.

We reproduced our analysis using those data and obtain results for 2013-2016 deforestation that are broadly consistent with the main results, but statistically insignificant (possibly because of the measurement error introduced when combining both datasets). They are presented in Table A.12.

Table A.12 – Deforestation by land use type, FACET

	All Forest		Primary Forest		Secondary Forest	
	(1) 2013-2016	(2) 2014	(3) 2013-2016	(4) 2014	(5) 2013-2016	(6) 2014
Lottery without truck	0.007	-0.136	0.133	-0.002	-0.130	-0.199
	(0.251)	(0.177)	(0.152)	(0.069)	(0.244)	(0.173)
	[0.978]	[0.443]	[0.385]	[0.980]	[0.596]	[0.252]
Lottery with truck	-0.099	-0.132	0.043	-0.008	-0.173	-0.165
	(0.230)	(0.162)	(0.139)	(0.064)	(0.224)	(0.158)
	[0.669]	[0.418]	[0.760]	[0.905]	[0.441]	[0.301]
Strat Vars	Yes	Yes	Yes	Yes	Yes	Yes
Observations	92	92	92	92	92	92
Mean Control	1.68	0.90	0.59	0.18	1.46	0.79
**P-v Test of Joint Significance**	0.82	0.36	0.49	0.93	0.45	0.20

* $p < 0.1$, ** $p < 0.05$, *** $p < 0.01$.

Source: Follow up surveys wave 2014, [2] and FACET

Note: Village level regressions use the average of two estimates of village-level deforestation per household, the first based on the aggregation of grid cells within the approximate boundaries of the village; and the second based on the aggregation of grid cells within a 5 km radius from the center of the village. Only pixels classified as either primary or secondary forest in FACET are considered. Columns 1, 3 and 5 show aggregate deforestation between 2013 and 2016, while columns 2, 4 and 6 show deforestation of 2014 to facilitate comparison with household level data. For all outcomes the inverse hyperbolic sine transformation is used.

OLS regression, robust standard errors in parentheses. P-values in square brackets. All regressions control for a full set of strata fixed effects. For all outcomes the inverse hyperbolic sine transformation is used (see Supplementary Material). Statistical tests are two-sided t-tests. The P-value of the F-test of joint significance of all treatment variables reported in the bottom part of the Table.

A.2.1 Randomization Inference

Because of the relatively small number of villages (92) and the multiple treatment variations, we also replicate all hypothesis tests using randomization-based inference tests [4,5]. In randomization-based inference, uncertainty in the estimates arises from the random assignment of the treatments rather than from sampling. This method allows estimating the exact p-value under the sharp null hypothesis that all treatment effects are zero by calculating all possible realizations of a test statistic and rejecting if the observed realization in the experiment itself is above the significance level cutoff for the generated distribution of test statistics. Randomization-based inference provides exact finite sample test statistics without appealing to asymptotic results and as such allows testing for the influence of potential outliers and protects against accidental imbalance affecting the results. Tables A.13 to A.17 provide these exact P-values for the estimates corresponding to Tables A.1 to A.3. The exact P-values lead to similar conclusions for all main results. The last line of each table indicate the P-values of the Wald omnibus test accounting the multiple experimental variations, as well as for the joint significance of the different coefficients within and across equations, following [4].

Table A.13 – Randomization Inference, Table A.1, part 1

	randomization c P-value	randomization t P-value
1 **Deforestation, 2014**		
Lottery without truck	.309	.305
Lottery with truck	.088	.09
Equation	.225	.225
2 **Deforestation, 2013-2016**		
Lottery without truck	.968	.968
Lottery with truck	.347	.347
Equation	.606	.582
	max c P-value	randomization c P-value
Omnibus test	.073	.073

Source: Follow up surveys wave 2014

Note: All regressions control for a full set of strata fixed effects. Omnibus test in the bottom of the table accounts for multiple comparisons.

Table A.14 – Randomization Inference, Table A.1, part 2

	randomization c P-value	randomization t P-value
1 **Forest, Season A 2014**		
Lottery without truck	.818	.813
Lottery with truck	.428	.432
Equation	.724	.738
2 **Primary Forest, Season A 2014**		
Lottery without truck	.004	.006
Lottery with truck	.097	.067
Equation	.02	.019
3 **Secondary Forest, Season A 2014**		
Lottery without truck	.213	.218
Lottery with truck	.952	.952
Equation	.02	.019
	max c P-value	randomization c P-value
Omnibus test	.048	.048

Source: Follow up surveys wave 2014

Note: Randomization inference with clustering at the village level. All regressions control for a full set of strata fixed effects. Omnibus test in the bottom of the table accounts for multiple comparisons.

Table A.15 – Randomization Inference, Table A.2 part 1

	randomization c<br>P-value	randomization t<br>P-value
1<br>**Total area farmed, Forest Season A 2014**		
Voucher low x no truck	.734	.697
Voucher high x no truck	.919	.922
Voucher low x truck	.744	.759
Voucher high x truck	.299	.271
Equation	.816	.767
2<br>**Total area farmed, Primary Forest, Season A 2014**		
Voucher low x no truck	.051	.028
Voucher high x no truck	.003	.024
Voucher low x truck	.083	.085
Voucher high x truck	.194	.117
Equation	.064	.081
3<br>**Total area farmed, Secondary Forest, Season A 2014**		
Voucher low x no truck	.562	.536
Voucher high x no truck	.137	.145
Voucher low x truck	.488	.481
Voucher high x truck	.662	.652
Equation	.376	.381
4<br>**Total area farmed, Fallow, Season A 2014**		
Voucher low x no truck	.262	.242
Voucher high x no truck	.604	.642
Voucher low x truck	.129	.125
Voucher high x truck	.818	.804
Equation	.071	.123

Source: Follow up surveys wave 2014

Note: Randomization inference with clustering at the village level. All regressions control for a full set of strata fixed effects. Omnibus test in the bottom of the table accounts for multiple comparisons.

Table A.16 – Randomization Inference, Table A.2 Part 2

	randomization c P-value	randomization t P-value
5<br>**Total area farmed, Savanna or other**		
Voucher low x no truck	.03	.129
Voucher high x no truck	.092	.191
Voucher low x truck	.558	.451
Voucher high x truck	.584	.551
Equation	.383	.649
6<br>**Total area farmed, Cultivated, Season A 2014**		
Voucher low x no truck	.166	.173
Voucher high x no truck	.743	.747
Voucher low x truck	.324	.336
Voucher high x truck	.503	.491
Equation	.142	.214
7<br>**Total area farmed, Season A 2014**		
Voucher low x no truck	.456	.417
Voucher high x no truck	.811	.822
Voucher low x truck	.263	.295
Voucher high x truck	.404	.359
Equation	.142	.214
	max c P-value	randomization c P-value
Omnibus test	.184	.183

Source: Follow up surveys wave 2014

Note: Randomization inference with clustering at the village level. All regressions control for a full set of strata fixed effects. Omnibus test in the bottom of the table accounts for multiple comparisons.

Table A.17 – Randomization Inference, Table A.3

	randomization c P-value	randomization t P-value
1 **Total agriulture wage labor**		
Voucher low x no truck	.563	.602
Voucher high x no truck	.748	.784
Voucher low x truck	.087	.064
Voucher high x truck	.5253993	.5164083
Equation	.549	.497
2 **Number of HH members working on farm**		
Voucher low x no truck	.0287747	.0177857
Voucher high x no truck	.0527684	.0307904
Voucher low x truck	.0170529	.0260439
Voucher high x truck	.0585499	.0665419
Equation	.099	.09
3 **Total labor sharing for land preparation**		
Voucher low x no truck	.2920444	.2680684
Voucher high x no truck	.100022	.093029
Voucher low x truck	.0314507	.0514307
Voucher high x truck	.557272	.500329
Equation	.168	.25
	max c P-value	randomization c P-value
Omnibus test	.157	.156

Source: Follow up surveys wave 2014

Note: Randomization inference with clustering at the village level. All regressions control for a full set of strata fixed effects. Omnibus test in the bottom of the table accounts for multiple comparisons.

A.3 Supplementary notes

The original modules (in French) of the household-level questionnaires used during the 2014 data collection are below.

Module C was used to collect information about every parcel cultivated by the household, including information on the size and location of the parcel, all the crops cultivated (seasonal, permanent, and horticulture crops), whether any improved seeds were used (for each seasonal crop) as well as other characteristics of the parcel . For each parcel, the year in which the field was converted is asked, as well as the prior land use on that land (providing the information on different types of land conversion in this paper).

Module E collects detailed information on labor used for plot preparation and cleaning, asking for different types of labor who worked, and how much time (days and hours) each worked. This allows to quantify the household and total labor used for land preparation, the number of household members working on the plot as well as wage income.

Section C. Champs du ménage A 2014 (1/4): ADMINISTRER AU CHEF DE MENAGE

Sur la base du croquis, et en respectant les numéros des champs: Listez tous les champs avec des cultures en Saison A 2014 (en incluant les champs en semi-jachère mais avec des cultures comme la banane ou le manioc en saison A 2014. Procédez ensuite horizontalement pour chaque champ, de la question C.4 à la question C.22.

<table>
<tr><td rowspan="2">id champ</td><td colspan="2">Quelle est la taille du champ?</td><td>Comment avez vous calculé cette taille?</td><td colspan="2">A quelle distance de ta maison se trouve ce champ?</td><td>Y avait-t-il des cultures vivrières (comme par exemple le mais, le riz, l'arachide ou le manioc) cultivées sur ce champ <u>en saison A 2014</u>, c'est à dire semées depuis janvier dernier et avant les vacances scolaires?</td><td colspan="5">Quelles cultures vivrières sont cultivées sur ce champ en saison A 2014?
Lister les 5 spéculations principales</td><td rowspan="2">Autre(s)</td><td>Si il y a du manioc, a-t-il été planté en saison A 2014?</td><td>S'il y avait au moins une des spéculations 1 à 6 sur ce champ, poser la question ; sinon, passez en C.7
Sur ce champ, toi ou un membre de ton ménage a-t-il utilisé des semences ou des boutures ameliorées pour au moins l'une des spéculations 1 à 6 <u>en saison A 2014</u>?</td></tr>
<tr><td>m^2</td><td>hectares</td><td>1= avec corde (Singa/ décamètre)
2=nombre de pas
3=nombre d'écart de bras
4=mesuré par quelqu'un d'autre
5=autre (preciser)
……………………….
……………………….</td><td>km</td><td>mètres</td><td>1=Oui
2=Non ▶ C.7</td><td colspan="5">1=Maïs
2=Riz
3=Arachide
4=Soja
5=Niébé
6=Manioc
7=Tarot
8=Patate douce
10=Haricot
11=Igname
12=Sésame
13=Ananas
14=Canne à sucre
15=Autre (préciser en C.5.b)</td><td>1=Oui
2=Non</td><td>1=Oui
2=Non
3=Ne sait pas ce qu'est une semence améliorée</td></tr>
<tr><td rowspan="2">id_a</td><td rowspan="2">C.1.a</td><td rowspan="2">C.1.b</td><td rowspan="2">C.2</td><td colspan="2">C.3</td><td rowspan="2">C.4</td><td colspan="5">C.5.a</td><td rowspan="2">C.5.b</td><td rowspan="2">C.5.c</td><td rowspan="2">C.6</td></tr>
<tr><td>a</td><td>b</td><td>1</td><td>2</td><td>3</td><td>4</td><td>5</td></tr>
<tr><td>1</td><td>|__||__||__| x |__||__||__|</td><td>|__||__|,|__||__|</td><td></td><td>|__||__|</td><td>|__||__||__|</td><td></td><td></td><td></td><td></td><td></td><td></td><td></td><td></td><td></td></tr>
<tr><td>2</td><td>|__||__||__| x |__||__||__|</td><td>|__||__|,|__||__|</td><td></td><td>|__||__|</td><td>|__||__||__|</td><td></td><td></td><td></td><td></td><td></td><td></td><td></td><td></td><td></td></tr>
<tr><td>3</td><td>|__||__||__| x |__||__||__|</td><td>|__||__|,|__||__|</td><td></td><td>|__||__|</td><td>|__||__||__|</td><td></td><td></td><td></td><td></td><td></td><td></td><td></td><td></td><td></td></tr>
<tr><td>4</td><td>|__||__||__| x |__||__||__|</td><td>|__||__|,|__||__|</td><td></td><td>|__||__|</td><td>|__||__||__|</td><td></td><td></td><td></td><td></td><td></td><td></td><td></td><td></td><td></td></tr>
<tr><td>5</td><td>|__||__||__| x |__||__||__|</td><td>|__||__|,|__||__|</td><td></td><td>|__||__|</td><td>|__||__||__|</td><td></td><td></td><td></td><td></td><td></td><td></td><td></td><td></td><td></td></tr>
<tr><td>6</td><td>|__||__||__| x |__||__||__|</td><td>|__||__|,|__||__|</td><td></td><td>|__||__|</td><td>|__||__||__|</td><td></td><td></td><td></td><td></td><td></td><td></td><td></td><td></td><td></td></tr>
<tr><td>7</td><td>|__||__||__| x |__||__||__|</td><td>|__||__|,|__||__|</td><td></td><td>|__||__|</td><td>|__||__||__|</td><td></td><td></td><td></td><td></td><td></td><td></td><td></td><td></td><td></td></tr>
<tr><td>8</td><td>|__||__||__| x |__||__||__|</td><td>|__||__|,|__||__|</td><td></td><td>|__||__|</td><td>|__||__||__|</td><td></td><td></td><td></td><td></td><td></td><td></td><td></td><td></td><td></td></tr>
<tr><td>9</td><td>|__||__||__| x |__||__||__|</td><td>|__||__|,|__||__|</td><td></td><td>|__||__|</td><td>|__||__||__|</td><td></td><td></td><td></td><td></td><td></td><td></td><td></td><td></td><td></td></tr>
<tr><td>10</td><td>|__||__||__| x |__||__||__|</td><td>|__||__|,|__||__|</td><td></td><td>|__||__|</td><td>|__||__||__|</td><td></td><td></td><td></td><td></td><td></td><td></td><td></td><td></td><td></td></tr>
</table>

Section C. Champs du ménage A 2014 (2/4) ADMINISTRER AU CHEF DE MENAGE

Poursuivre avec les champs de la page précédente

id champ	Y avait-t-il des cultures pérennes comme des bananes plantains, du café, du cacao, des arbres fruitiers ou des palmiers sur ce champ en saison A 2014?<br>1=Oui<br>2=Non ► C.9	Quelles cultures pérennes sont présentes sur ce champ en saison A 2014?<br>Lister les 3 principales<br>1=bananes<br>2= bananes plantains<br>3=café<br>4=cacao<br>5=palmiers<br>6=arbres fruitiers<br>7=autre (préciser en C.8.b)			Autre(s)	Y avait-t-il des cultures maraîchères (comme par exemple des légumes ou des piments) sur ce champ en saison A 2014, c'est à dire depuis janvier dernier et avant les vacances scolaires?<br>1=Oui<br>2=Non ► C.11	Quelles cultures maraîchères sont cultivées sur ce champ en saison A 2014?<br>Lister les 3 principales<br>1=oignon<br>2=tomate<br>3=ciboule<br>4=amarante<br>5=épinard<br>6=chicorée amère (bilolo)<br>7=aubergine<br>8=céleri<br>9=courge<br>10=gombo<br>11=piment<br>12=autre (préciser en C.10.b)			Autre(s)	En quelle année toi ou un membre de ton ménage a-t-il ouvert ou repris ce champ pour la dernière fois?<br>année	En quelle année ce champ a-t-il été ouvert pour la toute première fois?<br>1=2014<br>2=2013<br>3=2012<br>4=2011<br>5=2010<br>6=2009<br>7=2008<br>8=2007<br>9=Entre 2002 et 2007<br>10=Entre 1997 et 2002<br>11=Avant 1997	Avant [rep. C.12], y avait-il de la forêt primaire, de la forêt secondaire, ou de la jachère sur ce champ?<br>1=forêt primaire ► C.15<br>2=forêt secondaire► C.15<br>3=jachère<br>4=savanne► C.15<br>5=plantation► C.15<br>6=autre (préciser)► C.15<br>......................................<br>..................................	Avant [rep C.12], combien de temps avait duré la dernière jachère ?<br>1=1 an<br>2=2 ans<br>3=3 ans<br>4=4 ans<br>5=5 ans<br>6=6 ans<br>7=7 ans<br>8=8 ans<br>9=9 ans<br>10=10ans ou plus
id_a	C.7	C.8.a			C.8.b	C.9	C.10.a			C.10.b	C.12	C.12.2	C.13	C.14
		1	2	3			1	2	3					
1														
2														
3														
4														
5														
6														
7														
8														
9														
10														

Poursuivre avec les champs de la page précédente

id champ	Comment toi ou un membre de ton ménage a-t-il obtenu ce champ?<br>1=défrichage d'une terre qui n'avait jamais été cultivée avant vous (défrichage donne propriété)▶C.17<br>2=défrichage d'une terre qui appartenait à votre famille qui vous a été donnée/allouée par le chef de terres de la famille ou par un membre de votre famille▶C.17<br>3=Héritage (partage) suite au décès du chef des terres ou de votre père ▶C.17<br>4=prêt/confiage de la famille ▶C.17<br>5= confiage par la famille de la femme▶C.17<br>6=confié par un "non" parent ▶C.17<br>7=acheté<br>8=loué<br>9=don de la famille de la femme▶C.17<br>10=reçu comme compensation pour paiement d'arriérés?▶C.17<br>11=autre (préciser) ▶C.17<br>..	Quel est le prix de d'achat ou le prix de location par an?<br>**Si le contrat de location dure plus d'un an: diviser le montant total payé par le nombre d'années du contrat. Si terre achetée avant 2013, inscrire "0".**<br>montant	Unité<br>1=FC<br>2=$	Valeur en nature	Est-ce une terre de paysannat?<br>1=oui<br>2=non	Quelle est la couleur de la plus grande partie du sol de ce champ?<br>1=noir<br>2=rouge<br>3=blanc<br>4=autres<br>........................	A l'heure actuelle, y-a-t-il beaucoup, un peu, ou pas du tout de racines, de souches ou de termitières sur ce champ?<br>1=beaucoup<br>2=un peu<br>3=pas du tout	Considères-tu que ce champ est actuellement:<br>1= très fertile<br>2= fertile<br>3=moyen<br>4=pauvre<br>5= très pauvre	Toi ou quelqu'un de ton ménage possède-t-il un document qui vous donne des droits pour ce champ?<br>1=Oui<br>2=Non▶ **C.19.5**	Quel type de document?<br>1=droit d'usage (autorisation d'occupation) du chef de groupement<br>2=droit d'usage du chef du secteur<br>3=droit paysannat<br>4=document de décompte final<br>5=acte de vente<br>6= autre. Specifier<br>........................	Est-ce que votre champ est délimité?<br>1=oui, délimité par quelqu'un du ménage<br>2= oui, délimité par quelqu'un d'autre<br>3=non	Pour ce champ, as-tu la possibilité de le donner un jour à tes enfants ?<br>1=Oui<br>2=Non▶ **C.19.22**
id_a	C.15	C.16 a	C.16 b	C.16 c	C.17	C.18	C.19	C.19.2	C.19.3	C.19.4	C.19.10 a	C.19.19
1		|__||__||__||__||__|										
2		|__||__||__||__||__|										
3		|__||__||__||__||__|										
4		|__||__||__||__||__|										
5		|__||__||__||__||__|										
6		|__||__||__||__||__|										
7		|__||__||__||__||__|										
8		|__||__||__||__||__|										
9		|__||__||__||__||__|										
10		|__||__||__||__||__|										

Section C. Champs du ménage A 2014 (4/4) **ADMINISTRER AU CHEF DE MENAGE**

Poursuivre avec les champs de la page précédente

id champ	**Si le champ a été emblavé en saison A 2014**<br>Quelles sont les femmes responsables de l'entretien (sarclage), pour tout ou partie de ce champ en saison A 2014?<br><br>- Si 2 femmes ou plus sont citées pour le même champ, demander si le champ est divisé entre ces femmes.<br>- Si le champ est divisé, inscrire tous les cp des femmes responsables de l'entretien.<br>- Si le champ n'est pas divisé, demander qui est la responsable principale.<br>- Si il n'y a pas de femme responsable, inscrire le cp de la femme qui fait la plus grande partie du travail d'entretien<br>-Si il n'y a pas de femme qui fait le travail d'entretien inscrire le cp de l'homme responsable de l'entretien<br>-S'il n'y a pas eu d'entretien inscrire 88				**Indiquez avec "X" si des semences ameliorées de spéculations 1 à 5 ont été utilisées en saison A 2014 sur ce champ (voir C.6)**	**Indiquez avec "X" si des boutures ameliorées de manioc ont été utilisées en saison A 2014 sur ce champ (voir C.6)**	**Indiquez avec "X" si au moins une des spéculations 1 à 5 été semée en saison A 2014 sur ce champ et qu'il n'y a ni variétés ni boutures améliorées(voir C.5.a et C6)**	**Indiquez avec "X" s'il y a des boutures de manioc dont le semis a commencé en saison A 2014 sur ce champ, mais pas de spéculations 1 à 5 ni de variétés et boutures améliorées (voir C.5.a, C5c, C6)**
	cp	cp	cp	cp				
	C.20							
id_a	a	b	c	d	C.22	C.22.0	C.22.1	C.22.2
1								
2								
3								
4								
5								
6								
7								
8								
9								
10								

CP des répondants _____, _____, _____CP des autres membres présents _____, _____, _____

Section E: Main d'oeuvre pour la préparation du champ: pour tous les champs dans le tableau en section D - saison A 2014 (1/2)

Pour tous les champs de la section D, remplir une ligne par tâche (3 lignes par champ)

Tableau 1 : Main d'oeuvre familiale et gratuite (Gratuite=aucun paiement en nature, même un repas)													Tableau 2 : Ristournes de travail et associations										
Id Champ	**Tâche (une ligne par tâche)**	**Cette tâche a-t-elle été effectuée sur ce champ ?**	Combien de membres du ménage ont travaillé pour le [tâche] sur ce champ (hors ristourne)?	Qui sont les membres de votre ménage qui ont travaillé pour cette tâche? (hors ristourne) **Insérer le code de chaque personne**				Combien d'autres personnes ont travaillé pour cette tâche, sans être rémunérées?	*Si E1 et E3 = 0, passer a E6* Combien de temps a pris le travail effectué par la main d'oeuvre familiale ou non-rémunérée pour cette tâche?		Combien de jours par semaine ont-ils travaillé en moyenne pendant cette période ?	Combien d'heures par jours ont-ils travaillé ?	Combien de membres du ménage ont travaillé en tant que membres d'une ristourne de travail pour cette tâche?	Quels sont les membres du ménage qui ont travaillé en tant que membres de cette ristourne pour cette tâche? **Insérer le code de chaque personne**				Combien de personnes ont participé en tant que membres à la ristourne pour cette tâche au total?	Combien de temps a pris le travail de la ristourne sur ce champ pour cette tâche?		Combien de jours par semaine les membres de la ristourne ont-ils travaillé sur ce champ en moyenne pendant cette période ?	Combien d'heures par jours les membres de la ristourne ont-ils travaillé?
									Nombre	Unité									Nombre	Unité		
	1=défrichage<br>2=abattage<br>3=labour	1=oui<br>2=non ► tâche suivante	Nombre<br>Si 00, passer à E.3	cp	cp	cp	cp	Nombre		1=jour ► E.6<br>2=semaine<br>3=mois<br>4=autres (préciser)<br>......................	**Bien vérifier les jours de weekend**		Nombre<br>Si 00, passer à E.11	cp	cp	cp	cp	Nombre		1=jour ► E.10.2<br>2=semaine<br>3=mois<br>4=autres (préciser)<br>......................	**Bien vérifier les jours de weekend**	
id_a	tache	E.0	E.1	E.2 a	E.2 b	E.2 c	E.2 d	E.3	E.4 a	E.4 b	E.5	E.5.2	E.6	E.7 a	E.7 b	E.7 c	E.7 d	E.8	E.9 a	E.9 b	E.10	E.10.2
			|__||__|					|__||__|	|__||__|		|__|	|__||__|	|__||__|					|__||__|	|__||__|		|__|	|__||__|
			|__||__|					|__||__|	|__||__|		|__|	|__||__|	|__||__|					|__||__|	|__||__|		|__|	|__||__|
			|__||__|					|__||__|	|__||__|		|__|	|__||__|	|__||__|					|__||__|	|__||__|		|__|	|__||__|
			|__||__|					|__||__|	|__||__|		|__|	|__||__|	|__||__|					|__||__|	|__||__|		|__|	|__||__|
			|__||__|					|__||__|	|__||__|		|__|	|__||__|	|__||__|					|__||__|	|__||__|		|__|	|__||__|
			|__||__|					|__||__|	|__||__|		|__|	|__||__|	|__||__|					|__||__|	|__||__|		|__|	|__||__|
			|__||__|					|__||__|	|__||__|		|__|	|__||__|	|__||__|					|__||__|	|__||__|		|__|	|__||__|
			|__||__|					|__||__|	|__||__|		|__|	|__||__|	|__||__|					|__||__|	|__||__|		|__|	|__||__|
			|__||__|					|__||__|	|__||__|		|__|	|__||__|	|__||__|					|__||__|	|__||__|		|__|	|__||__|
			|__||__|					|__||__|	|__||__|		|__|	|__||__|	|__||__|					|__||__|	|__||__|		|__|	|__||__|
			|__||__|					|__||__|	|__||__|		|__|	|__||__|	|__||__|					|__||__|	|__||__|		|__|	|__||__|
			|__||__|					|__||__|	|__||__|		|__|	|__||__|	|__||__|					|__||__|	|__||__|		|__|	|__||__|
			|__||__|					|__||__|	|__||__|		|__|	|__||__|	|__||__|					|__||__|	|__||__|		|__|	|__||__|
			|__||__|					|__||__|	|__||__|		|__|	|__||__|	|__||__|					|__||__|	|__||__|		|__|	|__||__|
			|__||__|					|__||__|	|__||__|		|__|	|__||__|	|__||__|					|__||__|	|__||__|		|__|	|__||__|

Section E: Main d'oeuvre pour la préparation du champ: pour tous les champs dans le tableau de section D - saison A 2014 (2/2)

Poursuivre avec les champs de la page précédente.

Tableau 3. Main d'oeuvre rémunérée (y compris rémunération sous forme de repas)

Id Champ	**Tâche**	Combien de personnes ont travaillé sur ce champ en échange d'une rémunération (en espèces et/ou en nature) pour cette tâche?	Quelle rémunération (en FC ou en nature) toi ou quelqu'un de ton ménage a-t-il payé pour ce travail? (en moyenne par personne)				Est-ce que ces personnes ont beneficié d'un repas les jours de travail?	Pour combien de temps toi ou quelqu'un de ton ménage a-t-il embauché cette main d'oeuvre rémunérée pour cette tâche?		Combien de jours par semaine cette main d'oeuvre a-t-elle travaillé en moyenne pendant cette période ?	Combien d'heures par jours cette main d'œuvre a-t-elle travaillé?	Qui a payé la rémunération?	
								Nombre	Unité				
	1=défrichage<br>2=abattage<br>3=labour	Nombre<br>Si 00, ▶ prochaine tâche	Rémunération en FC par personne	1=par heure<br>2=par jour<br>3=par tâche	Rémunération en nature par personne (hors repas)	1=par heure<br>2=par jour<br>3=par tâche	1=Oui<br>2=Non		1=jour ▶ E.16<br>2=semaine<br>3=mois<br>4=autres (preciser)	Bien vérifier les jours de weekend		cp	cp
**id_a**	tache	E.11	E.12 a	E.12 b	E.12 c	E.12 d	E.13	E.14 a	E.14 b	E.15	E.15.2	E.16 a	E.16 b
		|__||__|	|__||__||__||__||__||__|					|__||__|		|__|	|__||__|		
		|__||__|	|__||__||__||__||__||__|					|__||__|		|__|	|__||__|		
		|__||__|	|__||__||__||__||__||__|					|__||__|		|__|	|__||__|		
		|__||__|	|__||__||__||__||__||__|					|__||__|		|__|	|__||__|		
		|__||__|	|__||__||__||__||__||__|					|__||__|		|__|	|__||__|		
		|__||__|	|__||__||__||__||__||__|					|__||__|		|__|	|__||__|		
		|__||__|	|__||__||__||__||__||__|					|__||__|		|__|	|__||__|		
		|__||__|	|__||__||__||__||__||__|					|__||__|		|__|	|__||__|		
		|__||__|	|__||__||__||__||__||__|					|__||__|		|__|	|__||__|		
		|__||__|	|__||__||__||__||__||__|					|__||__|		|__|	|__||__|		
		|__||__|	|__||__||__||__||__||__|					|__||__|		|__|	|__||__|		
		|__||__|	|__||__||__||__||__||__|					|__||__|		|__|	|__||__|		
		|__||__|	|__||__||__||__||__||__|					|__||__|		|__|	|__||__|		
		|__||__|	|__||__||__||__||__||__|					|__||__|		|__|	|__||__|		
		|__||__|	|__||__||__||__||__||__|					|__||__|		|__|	|__||__|		

CP des répondants _____, _____, _____CP des autres membres présents _____, _____, _____

A.4 Supplementary references

1. T. Bernard, S. Lambert, K. Macours, and M. Vinez. Adoption of Improved Seeds, Evidence from DRC. Working Paper 2019-72 (Paris School of Economics, 2019). [15]

2. M. C. Hansen, P. V. Potapov, R. Moore, M. Hancher, S. A. Turubanova, A. Tyukavina, D. Thau, S. V. Stehman, S. J. Goetz, T. R. Loveland, A. Kommareddy, A. Egorov, L. Chini, C. O. Justice, and J. R. G. Townshend. High-Resolution Global Maps of 21st-Century Forest Cover Change. *Science*, **342**, 850–853 (2013). ISSN 0036-8075, 1095-9203. doi: 10.1126/science.1244693. [3, 7, 10, 17, 19]

3. P. V. Potapov, S. A. Turubanova, M. C. Hansen, B. Adusei, M. Broich, A. Altstatt, L. Mane, and C. O. Justice. Quantifying Forest Cover Loss in Democratic Republic of the Congo, 2000–2010, with Landsat ETM+ Data. *Remote Sensing of Environment*, **122**, 106–116 (2012). ISSN 00344257. doi: 10.1016/j.rse.2011.08.027. [15]

4. A. Young. Channeling Fisher: Randomization Tests and the Statistical Insignificance of Seemingly Significant Experimental Results. *The Quarterly Journal of Economics*, **134**, 557–598 (2019). ISSN 0033-5533, 1531-4650. doi: 10.1093/qje/qjy029. [20]

5. S. Athey and G.W. Imbens. The Econometrics of Randomized Experiments. In Abhijit V. Banerjee and Esther Duflo, editors, *Handbook of Economic Field Experiments*, **1**, 73–140 (Elsevier, 2017). [20]
