# Supplementary material for: Impact of small farmers' access to improved seeds and deforestation in DR Congo
Source: Nat Commun. 2023 Mar 23;14:1603. doi: 10.1038/s41467-023-37278-2 (PMC10036623; doi:10.1038/s41467-023-37278-2)
Supplement: Supplementary file 2 — Reporting Summary [file 41467_2023_37278_MOESM2_ESM.pdf]

nature portfolio

Corresponding author(s): Karen Macours

Last updated by author(s): Nov 28, 2022

Reporting Summary

Nature Portfolio wishes to improve the reproducibility of the work that we publish. This form provides structure for consistency and transparency in reporting. For further information on Nature Portfolio policies, see our Editorial Policies and the Editorial Policy Checklist.

Statistics

For all statistical analyses, confirm that the following items are present in the figure legend, table legend, main text, or Methods section.

n/a	Confirmed	
☐	☒	The exact sample size (*n*) for each experimental group/condition, given as a discrete number and unit of measurement
☐	☒	A statement on whether measurements were taken from distinct samples or whether the same sample was measured repeatedly
☐	☒	The statistical test(s) used AND whether they are one- or two-sided<br>*Only common tests should be described solely by name; describe more complex techniques in the Methods section.*
☐	☒	A description of all covariates tested
☐	☒	A description of any assumptions or corrections, such as tests of normality and adjustment for multiple comparisons
☐	☒	A full description of the statistical parameters including central tendency (e.g. means) or other basic estimates (e.g. regression coefficient) AND variation (e.g. standard deviation) or associated estimates of uncertainty (e.g. confidence intervals)
☐	☒	For null hypothesis testing, the test statistic (e.g. *F*, *t*, *r*) with confidence intervals, effect sizes, degrees of freedom and *P* value noted<br>*Give P values as exact values whenever suitable.*
☒	☐	For Bayesian analysis, information on the choice of priors and Markov chain Monte Carlo settings
☒	☐	For hierarchical and complex designs, identification of the appropriate level for tests and full reporting of outcomes
☒	☐	Estimates of effect sizes (e.g. Cohen's *d*, Pearson's *r*), indicating how they were calculated

Our web collection on statistics for biologists contains articles on many of the points above.

Software and code

Policy information about availability of computer code

Data collection	no software used for data collection
Data analysis	Stata 14, Arcgis

For manuscripts utilizing custom algorithms or software that are central to the research but not yet described in published literature, software must be made available to editors and reviewers. We strongly encourage code deposition in a community repository (e.g. GitHub). See the Nature Portfolio guidelines for submitting code & software for further information.

Data

Policy information about availability of data

All manuscripts must include a data availability statement. This statement should provide the following information, where applicable:

- Accession codes, unique identifiers, or web links for publicly available datasets
- A description of any restrictions on data availability
- For clinical datasets or third party data, please ensure that the statement adheres to our policy

The data needed to reproduce the results are included in the submission and are available at https://www.openicpsr.org/openicpsr/project/177141. This includes all household and remote sensing data. FACET remote sensing data can also be access at https://carpe.umd.edu/carpemaps/. For Hansen et al remote sensing data see 10.1126/science.1244693.

Human research participants

Policy information about studies involving human research participants and Sex and Gender in Research.

Reporting on sex and gender	All analysis is at the household (or village) level, so no gender or sex disaggregation applies
Population characteristics	The population is representative of households in 92 villages spread across Sud Ubangi, Mongala and North Ubangi districts of the ex Equateur province, and across 5 (out of 9) territories in those districts.<br><br>More specifically: The RCT covered 92 villages (including control villages), for a total population of 223,069 individuals (including 26% adult men, 31% adult women, and 43% children), belonging to 29,792 households (of which 3% are female-headed households).<br><br>Within the treatment villages, 4394 households were randomly offered one discount voucher to access improved planting material at subsidized price. Amongst households elected to receive a voucher, the voucher recipient was an adult man in half of the cases, and an adult women in the other half.
Recruitment	A stratified random sample of households in treatment and control villages were selected for survey based on complete village listing (census) at baseline, and used in the present analysis. In each households, the household head was interviewed for the land-related survey modules on which the present study's results are based.<br><br>Informed consents were collected from all survey respondents. Compensation for survey time included 250g bars of soaps or 200g bags of salt. Non-response was very low (less than 10%), limiting concerns about selection bias.
Ethics oversight	The research received IRB approval from the ethics committee of PSE-JPAL Europe (reference number CE/2013-004). Informed consent was obtained from all human research participants.

Note that full information on the approval of the study protocol must also be provided in the manuscript.

Field-specific reporting

Please select the one below that is the best fit for your research. If you are not sure, read the appropriate sections before making your selection.

☐ Life sciences ☒ Behavioural & social sciences ☐ Ecological, evolutionary & environmental sciences

For a reference copy of the document with all sections, see nature.com/documents/nr-reporting-summary-flat.pdf

Behavioural & social sciences study design

All studies must disclose on these points even when the disclosure is negative.

Study description	quantitative experimental. Clustered Randomized Control Trial with 2 treatment arms and a control trial. Randomization of the type of subsidy at village level (over 92 villages). Randomization of the level of subsidy at the household level (within the 60 random treatment villages).
Research sample	The population is representative of households in 92 villages spread across Sud Ubangi, Mongala and North Ubangi districts of the ex Equateur province, and across 5 (out of 9) territories in those districts. The remote sensing analysis is done based on the area belonging to each of these 92 villages. Household level analysis is based on primary data collected from a stratified random sample of households in treatment and control villages. In each households, the household head was interviewed for the land-related survey modules on which the present study's results are based. 94% of the household heads are male, and they are on average 44 years old. The study sample of 92 villages chosen was selected among the larger set of villages targeted by PARRSA (the government intervention that is being evaluated) based on accessibility (i.e. the government's ability to reach the villages with truck delivery). The size and stratification of the household study sample (i.e. number of household surveyed in each village) was based on power calculations.
Sampling strategy	Stratified random, based on power calculations for experimental treatment effects of input subsidies
Data collection	Data collected with pen and paper by trained team of enumerators, who visited the 92 villages in teams, and did one-on-one in person interviews with the household heads of households in the sample. No other adult person was present during the interview. Enumerators were blind to the experimental treatment assignment and to the hypothesis tested in this paper.

Timing	Data collected between june 2014 and march 2015
Data exclusions	no data was excluded
Non-participation	2% of the sampled households could not be tracked at follow-up, due to migration outside of the region of study
Randomization	Randomization based on public lottery, organized in each of the treatment villages to which all sample households were invited. Village level randomization done at province level, in attendance of local authorities

Reporting for specific materials, systems and methods

We require information from authors about some types of materials, experimental systems and methods used in many studies. Here, indicate whether each material, system or method listed is relevant to your study. If you are not sure if a list item applies to your research, read the appropriate section before selecting a response.

Materials & experimental systems

n/a	Involved in the study
☒	☐ Antibodies
☒	☐ Eukaryotic cell lines
☒	☐ Palaeontology and archaeology
☒	☐ Animals and other organisms
☒	☐ Clinical data
☒	☐ Dual use research of concern

Methods

n/a	Involved in the study
☒	☐ ChIP-seq
☒	☐ Flow cytometry
☒	☐ MRI-based neuroimaging
